# Joint associations of social health and movement behaviours with mortality and cardiovascular disease: an analysis of 497,544 UK biobank participants

**DOI:** 10.1186/s12966-022-01372-3

**Published:** 2022-11-16

**Authors:** Karine Estelle Manera, Emmanuel Stamatakis, Bo-Huei Huang, Katherine Owen, Philayrath Phongsavan, Ben J Smith

**Affiliations:** 1 Sydney School of Public Health, Faculty of Medicine and Health, The Charles Perkins Centre, The University of Sydney, Sydney, Australia; 2 Sydney School of Health Sciences, Faculty of Medicine and Health , The Charles Perkins Centre, The University of Sydney, Sydney, Australia

**Keywords:** Physical activity, Sedentary behaviour, Loneliness, Isolation

## Abstract

**Background:**

Poor physical activity and excessive sedentary behaviour are well-established risk factors for morbidity and mortality. In the presence of emerging social problems, including loneliness and social isolation, these risks may be even greater. We aimed to investigate the joint effects of social health and movement behaviours on mortality and cardiovascular disease (CVD).

**Methods:**

497,544 UK Biobank participants were followed for an average of 11 years. Loneliness and social isolation were measured via self-report. Physical activity was categorised around current World Health Organisation (WHO) guidelines as low (< 600 metabolic equivalent of task [MET]-mins/week), moderate (600 < 1200) and high (≥ 1200). Sedentary behaviour was classified as low (≤ 3.5 h/day), moderate (3.5 ≤ 5) and high (> 5.5). We derived 24 social health–movement behaviour combinations, accordingly. Mortality and hospitalisations were ascertained to May 2020 for all-cause and CVD mortality, and non-fatal cardiovascular events.

**Results:**

Social isolation amplified the risk of both all-cause and CVD death across all physical activity and sedentary levels (hazard ratio, 95% confidence interval [HR, 95% CIs] for all-cause mortality; 1.58 [1.49 to 1.68] for low active-isolated vs. 1.26 [1.22 to 1.30] for low active-not isolated). Loneliness was only found to amplify the risk of death from cardiovascular disease among the high active and low sedentary participants. Loneliness and social isolation did not add to the risk of non-fatal cardiovascular events across most activity levels.

**Conclusion:**

The detrimental associations of poor physical activity and sedentary behaviour with mortality were consistently amplified by social isolation. Our study supports the need to target the socially isolated as a priority group in preventive public health strategies.

**Supplementary Information:**

The online version contains supplementary material available at 10.1186/s12966-022-01372-3.

## Background

Loneliness and social isolation are established social problems which have harmful impacts on mental and physical health [1–3]. Loneliness is a subjective experience characterised by a discrepancy in a person’s actual and desired level of social relationships [4], whereas social isolation is an objective and quantifiable indicator of a paucity of social contacts [5]. Loneliness and social isolation place individuals at increased risk of coronary heart disease and stroke [6]; and the burden of loneliness and isolation on mortality is comparable to the well-established risk factors of unhealthy behaviours, including physical inactivity and sedentary behaviour [7, 8].

A recent meta-analysis of data collected in 113 countries showed that loneliness is a common experience, particularly among adolescents and older adults [9]. This reported that the pooled prevalence of loneliness for adolescents (12–17 years) ranged from 9.2% in the South-East Asian region to 14.4% in the Eastern Mediterranean region; for young adults (18–29 years), the pooled estimates ranged from 1.8% in northern Europe to 9.4% in eastern Europe; for middle-aged adults (30–59 years), the estimates ranged from 2.7% in northern Europe to 18% in Central and Western Asia; among older adults (> 60 years), estimates ranged from 5.2 − 6.5% in northern Europe to 24.2% in eastern European countries. While equivalent regional estimates of the prevalence of social isolation are not available, national studies of persons aged 50 years and over have reported prevalence estimates ranging from 7.5% in Ireland [10], to 11% in the United States [11] and 17.3% in England [12].

The relationship of loneliness and isolation with mortality and cardiovascular morbidity is complex. Evidence suggests that these relationships are partly due to the associations between these negative social traits and unhealthy behaviours [13, 14]. Cross-sectional and longitudinal analyses have shown that lonely individuals are less likely to be physically active, and more likely to discontinue physical activity over time [15]. Social isolation is also associated with declines in physical activity and functioning [16, 17]. However, unhealthy behaviours may not entirely account for the relationship between these negative social traits and mortality. Mediation analyses of over 450,000 participants from the UK Biobank found that a cluster of health behaviours attenuated the relationship between loneliness and all-cause mortality, myocardial infarction and stroke by 41%, 35%, and 29%, respectively [13, 18]. The same cluster of health behaviours attenuated the deleterious associations of social isolation on mortality, myocardial infarction and stroke by 34%, 50% and 38%, respectively [13, 18].

As loneliness and isolation may impact health through mechanisms other than unhealthy behaviours, such as biological changes including increased cortisol secretion and reduced immune function [19, 20], individuals who display loneliness or social isolation in combination with physical inactivity/sedentary behaviour may have a compounded and higher risk of disease than those displaying only one of the risk factors. From a public health perspective, it is of interest to assess whether those who are active and lonely experience lower risk of death than those who are inactive and lonely. Given population studies which have found the prevalence of these exposures, particularly loneliness and physical inactivity, to differ between men and women[21, 22], it is possible that the consequences these jointly have for health outcomes may be moderated by sex.

Identifying the impact of these combined risk factors on major clinical outcomes is important to ensure interventions are targeting the most at-risk groups, however most existing evidence focuses on these risk factors in isolation [23, 24]. Further, there is growing interest in the synergistic benefits of addressing social connectedness and physical activity concurrently in interventions [25, 26], and it is valuable to explore whether there is an epidemiological rationale to adopt these approaches in policy and practice.

This study aimed to investigate the joint effects of loneliness and social isolation with physical inactivity and sedentary behaviour on all-cause mortality, cardiovascular disease (CVD) mortality, and non-fatal cardiovascular events.

## Methods

The UK Biobank is a prospective cohort study of 502,616 participants aged 40–69 years. Participants were recruited between 2006 and 2010 from 22 centres in the UK. A detailed description of the UK Biobank methods have been published elsewhere [27]. This study was conducted using the UK Biobank Resource under Application Number 25,813. All participants provided consent and de-identified data was provided to researchers for this analysis. We used the Strengthening the Reporting of Observational Studies in Epidemiology (STROBE) checklist to enhance the reporting of this study [28].

### Exposures

#### Loneliness and social isolation

Classifications for loneliness and social isolation were based on participant responses to self-report questions and were consistent with previous UK Biobank analyses [13, 18].

Loneliness was assessed with two questions: “*Do you often feel lonely?*” (yes = 1, no = 0) and “*How often are you able to confide in someone close to you?*” (1 = once every few months to never or almost never, 0 = almost daily to about once a month). Participants were classified as lonely if they had a summed score of 2 and not lonely if they had a summed score < 2.

Social isolation was assessed using three items: “*Including yourself, how many people are living together in your household?*” (living alone = 1, all other = 0); “*How often do you visit friends or family or have them visit you?*” (less than once a month = 1, once a month or more = 0); and “*Which of the following [leisure/social activities] do you engage in once a week or more often? You may select more than one*” (no participation in social activities at least weekly = 1, all other = 0). Participants were classified as socially isolated if they had a summed score of 2 or more and not socially isolated if they had a summed score < 2.

#### Physical activity and sedentary behaviour

Total physical activity was assessed using a modified version of the International Physical Activity Questionnaire (IPAQ) [29] which captures the frequency and duration of walking, moderate, and vigorous physical activity performed over the last seven days. In accordance with the IPAQ scoring protocol [30], total weekly physical activity (MET-mins/week) was calculated by multiplying the frequency and duration by the MET values. MET values include walking (3.3), moderate-intensity (4.0), and vigorous-intensity (8.0). Based on the current WHO guidelines [31], we grouped the sample into low active (< 600 MET-mins/week), moderate active (600 < 1200 MET-mins/week) and high active (≥ 1200 MET-mins/week) physical activity categories.

A sedentary behaviour variable was derived from three questions asking about the time participants spend per day watching TV, driving, and using a computer for non-work purposes: “*In a typical day, how many hours do you spend watching TV/driving/using a computer (not at work)?*“ We generated a continuous measure for total non-work sedentary behaviour time by summing the responses, and then categorised this into three groups based on tertiles (low sedentary behaviour ≤ 3.5 h/day, moderate sedentary behaviour 3.5 ≤ 5.5 h/day, and high sedentary behaviour > 5.5 h/day).

### Outcomes

Follow-up for all deaths was obtained from death certificates held by the National Health Service (NHS) Information Centre (England and Wales) and the NHS Central Register Scotland (Scotland). Hospital admissions were identified through data linkage to Hospital Admitted Patient Care Activity (England), General/Acute Inpatient and Day Case dataset (Scotland), and Patient Episode Database for Wales. Cause of death and hospital admissions were coded using the International Classification of Diseases 10th revision (ICD-10). Major non-fatal cardiovascular events were determined using the definition from Joshy et al. (Supplementary Table S1) [32], and CVD mortality was identified using this definition. Participants were followed from the date of attendance at the recruitment centre until May 2020. For the major non-fatal cardiovascular event outcome analyses, we excluded participants who previously experienced a major non-fatal cardiovascular event [32] prior to baseline to minimise the possibility of reverse causality.

### Covariates

We chose covariates that in previous literature have represented potential confounders in the relationship between social health and CVD/mortality, as well as movement behaviours and CVD/mortality [7, 13, 33, 34]: age, sex, ethnicity, area-level socioeconomic status, education (indicator of individual-level socioeconomic status [35]), alcohol consumption, smoking status, depressive symptoms and diabetes. A detailed description of covariates is provided in Supplementary Table S2.

For all-cause mortality outcome, additional covariates included pre-existing CVD and cancer (ICD-9/10 diagnosis and self-report). For the cardiovascular-related outcomes, additional covariates included body mass index (BMI), high blood pressure and high cholesterol. As physical activity and sedentary behaviour were correlated in our sample (Spearman’s rank correlation coefficient, *r*_*s*_ = -0.08, p < 0.001), as were loneliness and social isolation (*r*_*s*_ = 0.13, p < 0.001), we mutually adjusted where applicable i.e. where physical activity was an exposure, we included sedentary behaviour as a covariate; where loneliness was an exposure we included social isolation as a covariate, and vice versa [36].

### Statistical analysis

All analyses were performed using R software version 3.2.3 (R Foundation for Statistical Computing, Vienna, Austria). Descriptive statistics are presented as mean (standard deviation, SD) or number (percentage) for continuous and categorical variables, respectively. In the direct and joint effects analyses described below, we used Cox proportional hazard models where all-cause mortality and cardiovascular events were the outcomes, and we used the Fine-Gray competing-risks survival regression model where CVD mortality was the outcome [37]. Sensitivity analysis was conducted to determine the effects of missing data using multiple imputation. As this did not generate appreciable differences in the findings, the non-imputed data are reported.

We first examined the direct effects of loneliness and social isolation with all-cause mortality, CVD mortality and cardiovascular events using the not lonely and not isolated participants as the referent groups, respectively.

To examine the joint effects of loneliness and physical activity on all-cause mortality, CVD mortality and cardiovascular events, we derived a combined variable with six groups (combination of lonely/not lonely with low/moderate/high activity status). Those classified as high active and not lonely served as the reference group. The same groupings were done for loneliness and sedentary behaviour, social isolation and physical activity, and social isolation and sedentary behaviour, resulting in a total of 24 different groupings (Supplementary Tables S3and S4). The joint effects analyses include a crude model with no adjustments (Model 1), a minimally adjusted model including mutual adjustments for the alternate social health and movement behaviours (Model 2), and a fully adjusted model including all applicable covariates noted above (Model 3). Supplementary stratified analysis was conducted in which all models were stratified by gender.

#### Effects of interaction

We examined the effects of interaction on both the additive and multiplicative scales for the different pair-wise combinations of exposures (physical activity and loneliness, physical activity and isolation, sedentary behaviour and loneliness, and sedentary behaviour and isolation) using methods proposed by Källberg, Ahlbom [38]. Findings are presented as per recommendations by Knol and VanderWeele [39]. To generate dichotomous exposure variables for physical activity and sedentary behaviour as required for this analysis, we grouped physical activity levels into high (≥ 1200 MET-mins/week), and moderate/low (< 1200 MET-mins/week). We categorised sedentary behaviour into low (≤ 3.5 h/day), and moderate/high (> 3.5 h/day).

## Results

We excluded participants if they had missing data for both loneliness and social isolation, or physical activity and sedentary behaviour (n = 5072). For the major non-fatal cardiovascular event outcome analyses, 29,677 participants were excluded as they had previously experienced a major non-fatal cardiovascular event.

A total of 497,544 participants (56.5 ± 8.1 years old, 54% female) were included in the present study with an average follow-up time of 11.0 ± 1.5 years. Demographics characteristics of the sample are shown in Table 1.

**Table 1:** Descriptive statistics of study participants (n = 497,544)

Age, mean (SD)	56.5 (8.1)
Female, n (%)	270,738 (54.4)
Ethnicity, n (%)^a^	
White	470,028 (94.8)
Asian or Asian British	9347 (1.9)
Black or Black British	7765 (1.6)
Mixed	2924 (0.6)
Chinese	1503 (0.3)
Other Ethnic Group	4334 (0.9)
Socioeconomic Status, n (%)^a,b^	
Quintile 1 (least deprived)	171,533 (34.5)
2	106,898 (21.5)
3	82,642 (16.6)
4	75,867 (15.3)
Quintile 5 (most deprived)	59,985 (12.1)
Education, n (%)^c^	
College or University Degree	160,615 (32.8)
High School Diploma	219,159 (44.8)
Other/None	109,582 (22.4)
Alcohol Consumption, n (%)^a^	
Never	21,756 (4.4)
Previous Drinker	17,873 (3.6)
< 5 times/week	356,128 (71.6)
≥ 5 times/week	101,422 (20.4)
Cigarette Smoking, n (%)^a^	
Never	271,270 (54.7)
Previous Smoker	172,134 (34.7)
Current Smoker	52,379 (10.6)
Depressed Mood, n (%)^d^	
Not At All	363,102 (76.4)
Several Days or More	112,372 (23.6)
Unenthusiasm/Disinterest, n (%)^d^	
Not At All	377,158 (78.6)
Several Days or More	102,791 (21.4)
Tenseness/Restlessness, n (%)^d^	
Not At All	350,287 (73.3)
Several Days or More	127,328 (26.7)
Tiredness/Lethargy, n (%)^d^	
Not At All	224,836 (46.6)
Several Days or More	257,441 (53.4)
Pre-Existing Diabetes, n (%)^e^	26,012 (5.2)
Pre-Existing Cardiovascular Disease, n (%)^e^	41,184 (8.3)
Pre-Existing Cancer, n (%)^e^	41,839 (8.4)
Body Mass Index (kg/m2), n (%)^a^	
Underweight (< 18.5)	2591 (0.5)
Normal Weight (18.5–<25)	161,165 (32.6)
Overweight (25–<30)	210,327 (42.5)
Obese (30–<35)	86,640 (17.5)
Severely Obese (≥ 35)	34,222 (6.9)
High Blood Pressure, n (%)	119,094 (23.9)
High Cholesterol, n (%)	9178 (1.8)
Lonely, n (%)^d,f^	30,222 (6.4)
Socially Isolated, n (%)^c,g^	44,894 (9.2)
Physical Activity (MET-mins/week), n (%)^h^	
High (≥ 1200)	274,653 (61.7)
Moderate (600 < 1200)	79,552 (17.9)
Low (< 600)	90,756 (20.4)
Sedentary Behaviour (hours/day), n (%)^a^	
Low (≤ 3.5)	170,879 (34.4)
Moderate (3.5 ≤ 5.5)	180,645 (36.4)
High (> 5.5)	144,934 (29.2)

^a^ Missing data < 1%

^b^ The Townsend area deprivation index served as an indicator of socioeconomic status, with higher quintile scores indicating greater socioeconomic deprivation

^c^ Missing data < 2%

^d^ Missing data < 5%

^e^ Diagnosis based on ICD9/10 and self-reports

^f^ Loneliness was assessed based on responses to two questions: “Do you often feel lonely?” and “How often are you able to confide in someone close to you?”

^g^ Social isolation was assessed based on responses to three questions: “Including yourself, how many people are living together in your household?”; “How often do you visit friends or family or have them visit you?”; and “Which of the following [leisure/social activities] do you engage in once a week or more often?”

^h^ Missing data < 11%

### Direct effects of exposures with mortality and non-fatal CVD events

Direct effects analysis (Supplementary Tables S5a and S5b) showed that, compared to those who were not lonely, lonely individuals had an increased risk of CVD mortality (hazard ratio, HR, [95% confidence intervals]: 1.19 [1.07 to 1.32]), but not a higher risk of all-cause mortality (0.98 [0.94 to 1.03]). Participants who were socially isolated had higher risks of all-cause mortality (1.33 [1.28 to 1.38]) and CVD mortality (1.49 [1.37 to 1.62]) compared to those not isolated. Stratified analyses revealed that women who were socially isolated had a higher risk of non-fatal CVD events (1.06 [1.00-1.11]), whereas this was not the case among men.

Relative to high physical activity, moderate and low levels of physical activity were associated with higher risk of all-cause mortality (1.09 [1.05 to 1.13] for moderate; 1.24 [1.21 to 1.28] for low). Those with low physical activity also had higher risk of CVD mortality (1.23 [1.15 to 1.33]) and non-fatal CVD events (1.03 [1.00 to 1.06]). In addition, women undertaking moderate (compared with high) physical activity had an increased risk of CVD mortality (1.20 [1.01–1.41]). For sedentary behaviour, participants displaying moderate or high sedentary behaviour had greater risk of all-cause mortality (1.05 [1.01 to 1.08] for moderate; 1.08 [1.05–1.12] for high) and non-fatal CVD events (1.07 [1.04 to 1.10] for moderate; 1.09 [1.06 to 1.12] for high) compared to those with low levels. Stratified analyses showed that the elevated risk of all-cause mortality associated with moderate and high sedentary behaviour was significant for women, but not for men.

### Joint association of exposures with mortality and non-fatal CVD events

**Fig. 1:**
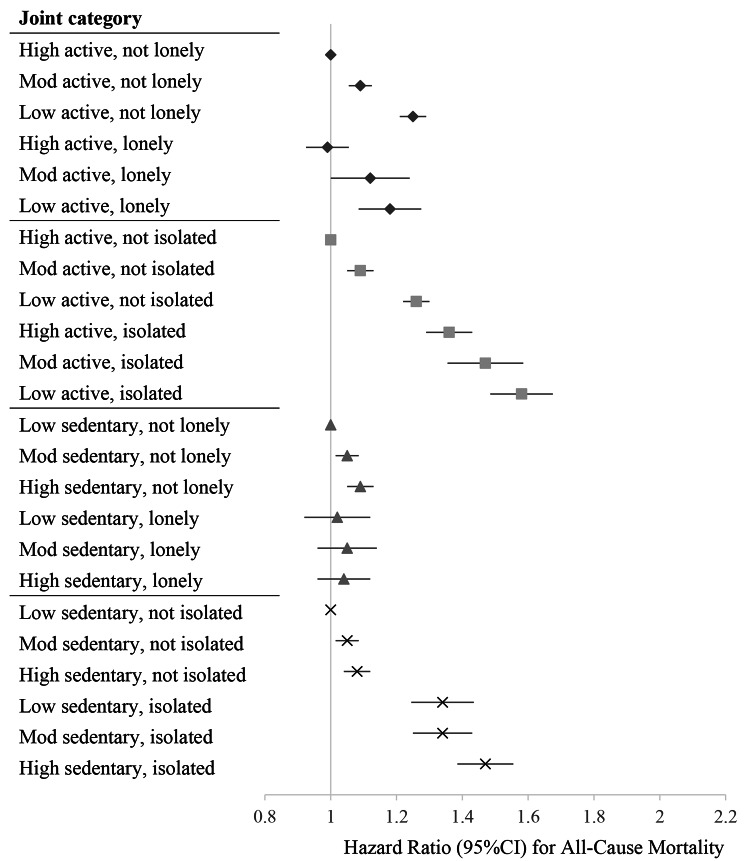
Joint associations of movement behaviour and social health status with all-cause mortality. *Note.* Physical activity levels were categorized based on public health guidelines: low active (< 600 MET-mins/week), moderate active (600 < 1200) and high active (≥ 1200 MET-mins/week). Sedentary behaviour was categorized into: low, ≤ 3.5 h/day; moderate, 3.5 ≤ 5.5 h/day; high > 5.5 h/day. Models were adjusted for age, sex, ethnicity, smoking status, alcohol consumption, education, socioeconomic status, depressive symptoms, diabetes, CVD and cancer. Mutual adjustments were made for physical activity, sedentary behaviour, loneliness and social isolation, when not included as an exposure

**Fig. 2:**
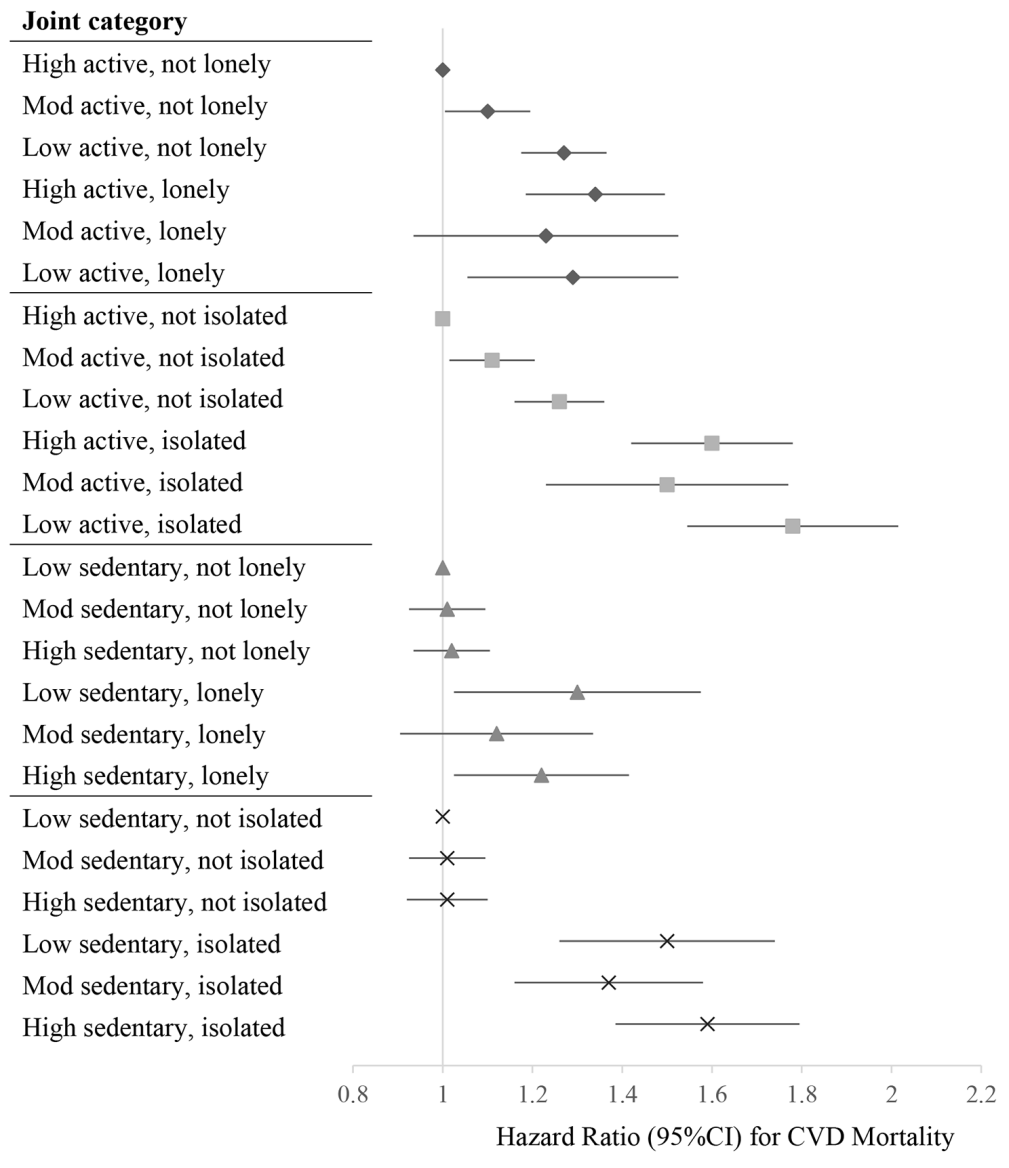
Joint associations of movement behaviour and social health status with CVD mortality. *Note.* Physical activity levels were categorized based on public health guidelines: low active (< 600 MET-mins/week), moderate active (600 < 1200) and high active (≥ 1200 MET-mins/week). Sedentary behaviour was categorized into: low, ≤ 3.5 h/day; moderate, 3.5 ≤ 5.5 h/day; high > 5.5 h/day. Models were adjusted for age, sex, ethnicity, smoking status, alcohol consumption, education, socioeconomic status, depressive symptoms, diabetes, hypertension, high cholesterol and BMI. Mutual adjustments were made for physical activity, sedentary behaviour, loneliness and social isolation, when not included as an exposure

**Fig. 3:**
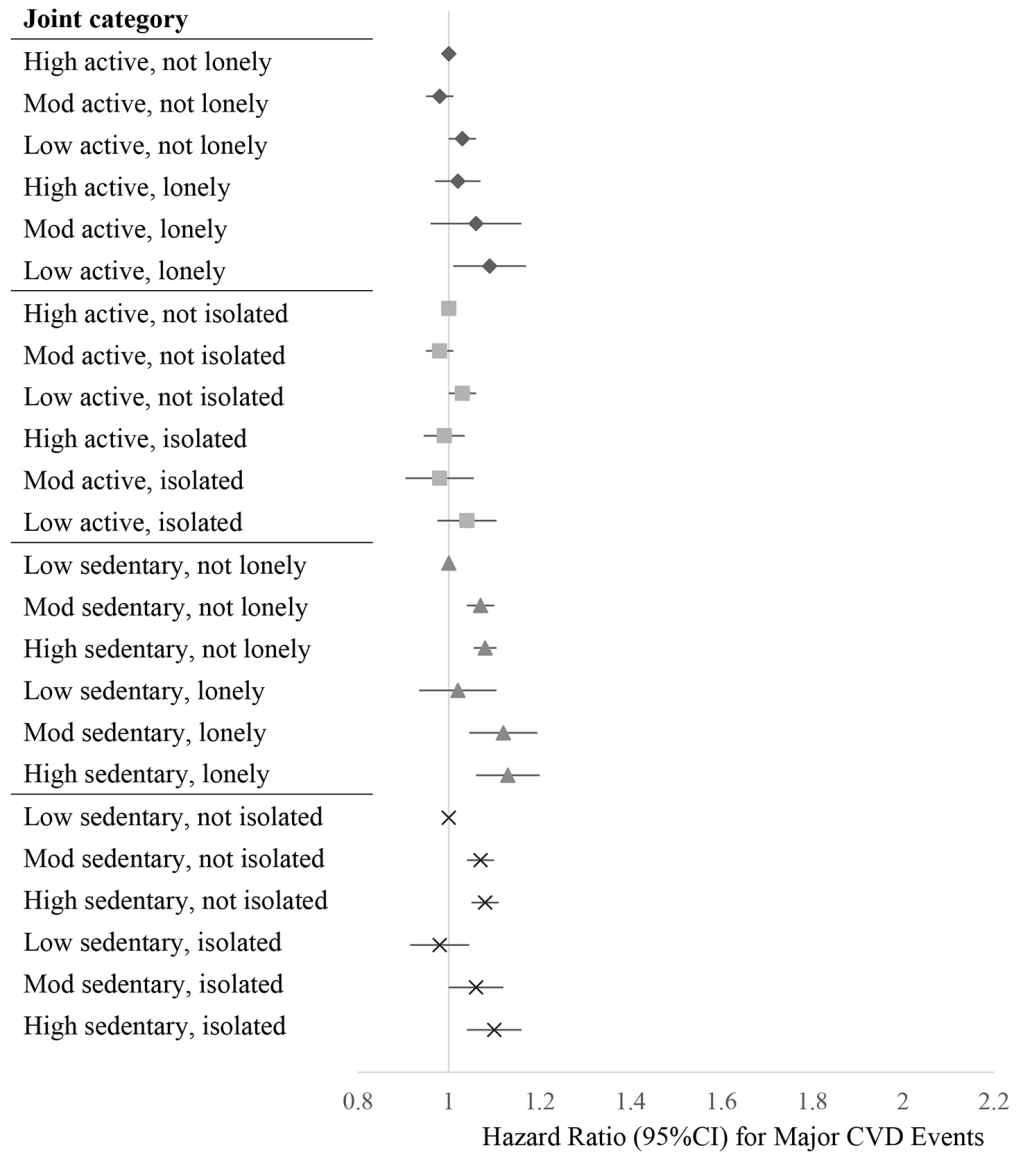
Joint associations of movement behaviour and social health status with major non-fatal cardiovascular events. *Note.* Physical activity levels were categorized based on public health guidelines: low active (< 600 MET-mins/week), moderate active (600 < 1200) and high active (≥ 1200 MET-mins/week). Sedentary behaviour was categorized into: low, ≤ 3.5 h/day; moderate, 3.5 ≤ 5.5 h/day; high > 5.5 h/day. Models were adjusted for age, sex, ethnicity, smoking status, alcohol consumption, education, socioeconomic status, depressive symptoms, diabetes, hypertension, high cholesterol and BMI. Mutual adjustments were made for physical activity, sedentary behaviour, loneliness and social isolation, when not included as an exposure

Figures 1, 2 and 3, and Supplementary Tables S6a-S8c  present the hazard ratios for each exposure combination compared to the referent high movement behaviour-healthy social status group. Supplementary Tables S6a-S8c  also report results from the crude and minimally adjusted models. The following text corresponds to the results from the fully adjusted models.

#### Physical activity and loneliness

Participants who undertook low or moderate levels of physical activity had an increased risk of all-cause mortality compared to those with high activity, however these effects did not differ based on whether a person was lonely or not (for low active-lonely, 1.18 [1.09 to 1.28]; for low active-not lonely, 1.25 [1.21 to 1.29]). For CVD mortality, the addition of loneliness did not further increase the risk among those with lower levels of physical activity, however it did increase the risk for those who were highly active (1.34 [1.17 to 1.54] for high active-lonely vs. high active-not lonely [reference]). For major non-fatal cardiovascular events, only low active participants had an increased risk, and this was slightly greater for those jointly expressing loneliness (for low active-not lonely, 1.03 [1.00 to 1.06]; for low active-lonely, 1.09 [1.01 to 1.17]). Stratified analyses showed that men who were moderately active and lonely had a higher risk of non-fatal cardiovascular events (1.16 [1.04–1.31]), whereas men who were moderately active and not lonely did not have this elevated risk.

#### Physical activity and social isolation

Compared with the reference group (high active-not isolated), all exposure combinations had higher risks of all-cause mortality. There was a dose-response increase in mortality risk with decreasing physical activity among both the isolated and not isolated. Additionally, the risk of death was greater in isolated individuals across all levels of physical activity compared to their non-isolated counterparts (low active-isolated, 1.58 [1.49 to 1.68] vs. low active-not isolated, 1.26 [1.22 to 1.30]; moderate active-isolated, 1.47 [1.36 to 1.59] vs. moderate active-not isolated, 1.09 [1.05 to 1.13]). For CVD mortality, all exposure combinations had a higher risk compared to the high active-not isolated group. The presence of social isolation further increased the risk of CVD mortality across all levels of activity (low active-isolated, 1.78 [1.56 to 2.03] vs. low active-not isolated, 1.26 [1.17 to 1.37]; moderate active-isolated, 1.50 [1.25 to 1.79] vs. moderate active-not isolated, 1.11 [1.02 to 1.21]). In stratified analyses it was found that among women the presence of social isolation did not significantly increase the risk of CVD mortality associated with low and moderate levels of physical activity. No differences were found among the exposure combinations compared to the reference group for major non-fatal cardiovascular events.

#### Sedentary behaviour and loneliness

Compared to the reference group (low sedentary-not lonely), we found an increased risk of all-cause mortality with higher sedentary behaviours among participants who were not lonely (for moderate sedentary-not lonely, 1.05 [1.01 to 1.08]; for high sedentary-not lonely, 1.09 [1.05 to 1.13]). Increased risk of CVD mortality was seen in the low sedentary-lonely (1.30 [1.05 to 1.60]) and high sedentary-lonely (1.22 [1.04 to 1.43]) groups. For major non-fatal cardiovascular events, there was a slight dose response effect with increasing sedentariness among both lonely and not lonely participants.

#### Sedentary behaviour and social isolation

Compared with the reference group (low sedentary-not isolated), all exposure combinations had higher risks of all-cause mortality, with the greatest being for high sedentary-isolated participants (1.47 [1.39 to 1.56]). Isolated participants had greater risks across all sedentary behaviour levels, compared to non-isolated participants. However, in stratified analyses it was found that women who were isolated and had low sedentariness did not have a higher risk of all-cause mortality. Across all sedentary behaviour levels, participants who were isolated had greater risk of CVD mortality, with the highest risk in those expressing both high sedentary behaviour and isolation (1.59 [1.39 to 1.80]). For major non-fatal cardiovascular events, increased risks were found among those not isolated and with moderate or high sedentary behaviour, however the greatest risk was among the high sedentary-isolated group (1.10 [1.04–1.16]).

#### Analysis of interaction

There was no evidence of interaction among any of the exposure combinations for all-cause mortality. For CVD mortality and non-fatal cardiovascular events, we found weak and inconsistent evidence of interaction within the physical activity and loneliness combination (Supplementary Table S9a-d).

## Discussion

Our results show that loneliness did not increase the risk of all-cause or CVD death among those physically inactive or more sedentary, but being socially isolated did. The same relationship was found among highly active and low sedentary participants for all-cause mortality. However, for CVD mortality, both loneliness and isolation increased the risk of CVD death among those highly active and low sedentary participants. Unlike the mortality outcomes, no consistent associations were seen between any of the exposure combinations and major non-fatal cardiovascular events.

We found that social isolation had a consistently greater impact on mortality than loneliness, regardless of one’s level of movement. This is supported by our analysis of the direct effects of loneliness and isolation on mortality, which found stronger associations with CVD mortality among the isolated compared to the lonely, and no association between loneliness and all-cause mortality. Our finding concerning the risk of all-cause mortality associated with loneliness (HR 0.98) contrast with those from a meta-analysis including 70 studies with over 3 million participants followed for 7 years, in which the adjusted odds ratio (AOR) of death was 1.26 in those classified as lonely [7]. This seminal work by Holt-Lunstad et al. found that both loneliness and social isolation resulted in a similar increased risk of death, and established loneliness and isolation as risk factors for mortality.

A key consideration when comparing across studies is the method of measurement for the social health exposures. Our study utilised the relevant available items in the UK Biobank, and is consistent and comparable with previous Biobank publications [13]. However, the two items comprising our loneliness scale have not been psychometrically validated, and may differ from the measures included in the meta-analysis by Holt-Lunstad et al., such as the De Jong Gierveld Loneliness Scale [40] or the University of Los Angeles (UCLA) Loneliness Scale [41]. This highlights the need for measurement standardisation to improve the comparability of data and enhance our understanding of these critical issues. It is also important to note that, unlike social isolation, loneliness is a cognitive-affective state and may be more prone to variation than objectively measured isolation [2]. Our measurement of loneliness at one point in time, as opposed to identifying stable or chronic loneliness, may be a limitation given the complexity of this condition.

Previous studies have suggested synergistic associations between social health and movement behaviours, however these have primarily assessed the independent effects of these exposures on health [42], as well as the bidirectional relationships that these exposures have with each other i.e. that loneliness and social isolation have a negative impact on physical activity behaviour, and that physical activity reduces loneliness [15, 43, 44]. To our knowledge, this is the first study investigating the joint effects of social health and movement behaviours on health outcomes. Our findings reveal that a combination of social isolation and low physical activity or high sedentariness is associated with the highest risk of mortality. This supports calls for consistent, standardised assessments of isolation to be adopted alongside those for established risk factors like physical inactivity in health care and other settings [45], in order to identify those who are a high priority for preventive care. Further, this provides a rationale for continuing to develop and test interventions that address these risk factors in tandem, such as group-based programs that support both regular physical activity and social connection. While we saw greatest risk of mortality among those with worst movement behaviour and social health, our tests for interaction for most of the exposure combinations were less clear. The statistically significant but inconsistent findings among the loneliness and physical activity combination for CVD mortality and non-fatal cardiovascular events suggests that further research is needed to elucidate the mechanism behind which these two exposures interact to jointly amplify cardiovascular health risks.

The strengths of the present study include the prospective study design with large sample size and long follow-up duration. Limitations include the use of self-reported measures of movement behaviours. While our physical activity measures examined frequency, duration and intensity, both over and under-estimation of total activity has been documented for self-report measures of this behaviour [46]. The sedentary behaviour measure was consistent with previous Biobank studies [47, 48] but does not include occupational sedentary behaviour, which may have led to exposure misclassification. However, sedentary behaviour shows strong socioeconomic patterning in the UK [49] (the higher the SES the higher total sedentary time) and we adjusted for both individual and area level socioeconomic status. The measures of loneliness and social isolation used in the UK Biobank study have not been validated, and generated prevalence estimates at the lower end of the range of those reported in previous population studies in the United Kingdom. This may be due to selection bias in the UK Biobank cohort, and a tendency for the classification method adopted in the analysis of loneliness and isolation in this cohort to identify those with more acute levels of these conditions. Nonetheless, the purpose of our study was to examine predictive associations, rather than the prevalence of loneliness and social isolation. Finally, the observational nature of our data limits any causal interpretation of our findings, however we did attempt to minimise confounding by adjusting for a wide range of covariates and excluding groups with existing conditions.

## Conclusion

In conclusion, we found that low levels of physical activity and higher levels of sedentary behaviour increased the risk of death, and this was consistently amplified by social isolation. The results of this study highlight the importance of both movement behaviours and social health, and that the socially isolated should be identified as a priority group. Future public health intervention research should take these factors into account, and evaluate the synergistic benefits of social interaction and movement to improve mental and physical health for those most vulnerable.

## Electronic supplementary material

Below is the link to the electronic supplementary material.

Supplementary Material 1

Supplementary Material 2

## Data Availability

The data that support the findings of this study are available from the UK Biobank but restrictions apply to the availability of these data, which were used under license for the current study, and so are not publicly available. Researchers will need to apply to access the UK Biobank database at the following link: https://www.ukbiobank.ac.uk/enable-your-research/apply-for-access.

## Abbreviations

BMI: Body mass index
CVD: Cardiovascular disease
HR: Hazard ratio
ICD-10: International Classification of Diseases 10th revision
IPAQ: International Physical Activity Questionnaire
MET: Metabolic equivalent of task
NHS: National Health Service
STROBE: Strengthening the Reporting of Observational Studies in Epidemiology
UCLA: University of California Los Angeles
WHO: World Health Organisation
