# Supplementary material for: Joint associations of social health and movement behaviours with mortality and cardiovascular disease: an analysis of 497,544 UK biobank participants
Source: Int J Behav Nutr Phys Act. 2022 Nov 16;19:137. doi: 10.1186/s12966-022-01372-3 (PMC9670497; doi:10.1186/s12966-022-01372-3)
Supplement: Supplementary file 2 — Supplementary Material 2 [file 12966_2022_1372_MOESM2_ESM.docx]

**Supplementary Files.**

**Supplementary Table S1.** Definition of major cardiovascular disease

**Supplementary Table S2.** Description of selected covariates

**Supplementary Table S3.** Number of mortality events by joint category of social health status and movement behaviour

**Supplementary Table S4.** Number of major non-fatal CVD events by joint category of social health status and movement behaviour

**Supplementary Table S5a.** Independent effects of physical activity, sedentary behaviour, loneliness and social isolation on all-cause and CVD mortality and CVD events

**Supplementary Table S5b.** Independent effects of physical activity, sedentary behaviour, loneliness and social isolation on all-cause and CVD mortality and CVD events stratified by sex

**Supplementary Table S5c.** Independent effects of physical activity, sedentary behaviour, loneliness and social isolation on all-cause and CVD mortality and CVD events with multiple imputation

**Supplementary Table S6a.** Joint associations of movement behaviour and social health status with all-cause mortality

**Supplementary Table S6b.** Joint associations of movement behaviour and social health status with all-cause mortality in men

**Supplementary Table S6c.** Joint associations of movement behaviour and social health status with all-cause mortality in women

**Supplementary Table S7a.** Joint associations of movement behaviour and social health status with CVD mortality

**Supplementary Table S7b.** Joint associations of movement behaviour and social health status with CVD mortality in men

**Supplementary Table S7c.** Joint associations of movement behaviour and social health status with CVD mortality in women

**Supplementary Table S8a.** Joint associations of movement behaviour and social health status with major non-fatal cardiovascular events

**Supplementary Table S8b.** Joint associations of movement behaviour and social health status with major non-fatal cardiovascular events in men

**Supplementary Table S8c.** Joint associations of movement behaviour and social health status with major non-fatal cardiovascular events in women

**Supplementary Table S9a.** Analyses on interaction of physical activity and loneliness with risks for all-cause mortality, CVD mortality, and major non-fatal cardiovascular disease

**Supplementary Table S9b.** Analyses on interaction of physical activity and isolation with risks for all-cause mortality, CVD mortality, and major non-fatal cardiovascular disease

**Supplementary Table S9c.** Analyses on interaction of sedentary behaviour and loneliness with risks for all-cause mortality, CVD mortality, and major non-fatal cardiovascular disease

**Supplementary Table S9d.** Analyses on interaction of sedentary behaviour and isolation with risks for all-cause mortality, CVD mortality, and major non-fatal cardiovascular disease

**Supplementary Table S1.** Definition of major cardiovascular disease, based on Joshy et al. (2015) (http://dx.doi.org/10.17061/phrp2531532)

| ICD-10 code | Description |
| --- | --- |
| Selected hypertensive diseases | |
| I11 | Hypertensive heart disease |
| I12 | Hypertensive kidney disease |
| I13 | Hypertensive heart and kidney disease |
| Ischaemic heart disease | |
| I20 | Angina pectoris |
| I21 | Acute myocardial infarction |
| I22 | Subsequent myocardial infarction |
| I23 | Certain current complications following acute myocardial infarction |
| I24 | Other acute ischaemic heart diseases |
| I25 | Chronic ischaemic heart disease |
| Pulmonary heart disease and diseases of pulmonary circulation | |
| I26 | Pulmonary embolism |
| I27 | Other pulmonary heart diseases |
| I28 | Other diseases of pulmonary vessels |
| Selected other forms of heart disease | |
| I34 | Nonrheumatic mitral valve disorders |
| I35 | Nonrheumatic aortic valve disorders |
| I36 | Nonrheumatic tricuspid valve disorders |
| I42 | Cardiomyopathy |
| I44 | Atrioventricular and left bundle-branch block |
| I46 | Cardiac arrest |
| I47 | Paroxysmal tachycardia |
| I48 | Atrial fibrillation and flutter |
| I49 | Other cardiac arrhythmias |
| I50 | Heart failure |
| I51 | Complications and ill-defined descriptions of heart disease |
| Selected cerebrovascular disease | |
| I61 | Intracerebral haemorrhage |
| I62 | Other nontraumatic intracranial haemorrhage |
| I63 | Cerebral infarction |
| I64 | Stroke, not specified as haemorrhage or infarction |
| I65 | Occlusion and stenosis of precerebral arteries, not resulting in cerebral infarction |
| I66 | Occlusion and stenosis of cerebral arteries, not resulting in cerebral infarction |
| I67 | Other cerebrovascular diseases |
| I69 | Sequelae of cerebrovascular disease |
| Selected diseases of arteries, arterioles and capillaries | |
| I70 | Atherosclerosis |
| I71 | Aortic aneurysm and dissection |
| I72 | Other aneurysm and dissection |
| I73 | Other peripheral vascular diseases |
| I74 | Arterial embolism and thrombosis |
| I77 | Other disorders of arteries and arterioles |
| Selected diseases of veins, lymphatic vessels and lymph nodes, not elsewhere classified | |
| I80 | Phlebitis and thrombophlebitis |
| Selected episodic and paroxysmal disorders | |
| G45 | Transient cerebral ischaemic attacks and related syndromes |
| G46 | Vascular syndromes of brain in cerebrovascular diseases |

**Supplementary Table S2.** Description of selected covariates (except for age and sex)

| Covariates | UK BioBank Code | Description | Category |
| --- | --- | --- | --- |
| Ethnicity | 21000 | The existing variable 'ethnic background' was applied. | White; Mixed; Asian or Asian British;  Black or Black British; Chinese; Other ethnic group |
| Socioeconomic status | 189 | The existing variable 'Townsend area deprivation index' served as an indicator of socioeconomic status, with higher scores indicating greater socioeconomic deprivation. We grouped scores into quintiles. | Quintile 1 (least deprived) to Quintile 5 (most deprived). |
| Education | 6138 | The existing variable 'qualifications' was applied. We further grouped the options into 3 categories. | College or University degree; High School Diploma; Other/none |
| Alcohol consumption | 1558, 20117 | Alcohol consumption was categorised using number of days alcohol was consumed per week. | Never; Previous; <5 times/week; ≥5 times/week |
| Cigarette smoking | 20116 | The existing variable 'smoking status' was applied. | Never; Previous smoker; Current smoker |
| Depressive symptoms | 2050, 2060, 2070, 2080 | Frequency of depressed mood, unenthusiasm / disinterest, tenseness / restlessness, and tiredness / lethargy in last 2 weeks. | Not at all; Several days; More than half the days; Nearly every day |
| Diabetes | 41270, 41271, 2443 | Before recruitment, diagnosed with all-type diabetes, based on both ICD9/10 and self-report. | Yes; No |
| Additional covariates for all-cause mortality | | |  |
| Cardiovascular disease | 41270,  41271, 6150 | Before recruitment, diagnosed with CVD based on Joshy et al 2016, based on both ICD9/10 and self-report. | Yes; No |
| Cancer | 41270,  41271, 2453 | Before recruitment, diagnosed with major cancer, based on both ICD9/10 and self-report. | Yes; No |
| Additional covariates for CVD mortality and major non-fatal CVD events | | |  |
| Body Mass Index (BMI) | 21001 | Value is constructed from height and weight measured during the initial Assessment Centre visit. We grouped this into 5 categories. | Underweight (<18.5kg/m2); Normal weight (18.5–<25 kg/m2); Overweight (25–<30 kg/m2);  Obese (30–<35 kg/m2); Severely obese (≥35 kg/m2). |
| High blood pressure | 6150 | Vascular/heart problems diagnosed by doctor: high blood pressure | Yes; No |
| High cholesterol | 20002 | Other serious medical condition/disability diagnosed by doctor: high cholesterol | Yes; No |

Further details of these measures can be found in the UK Biobank online data showcase https://biobank.ndph.ox.ac.uk/showcase/search.cgi

**Supplementary Table S3.** Number of mortality events by joint category of social health status and movement behaviour

| Joint Category ^a^ | N With/Without Events | |
| --- | --- | --- |
|  | All-cause mortality | CVD mortality |
| Physical Activity, Loneliness Status (n=427,781) | 25,023/402,758 | 4,700/423,081 |
| High, not lonely (n=249,993) | 13,142/236,851 | 2,327/247,666 |
| Moderate, not lonely (n=71,750) | 4,089/67,661 | 744/71,006 |
| Low, not lonely (n=79,620) | 5,752/73,868 | 1,145/78,475 |
| High, lonely (n=14,845) | 991/13,854 | 243/14,602 |
| Moderate, lonely (n=4,606) | 370/4,236 | 82/4,524 |
| Low, lonely (n=6,967) | 679/6,288 | 159/6,808 |
| Physical Activity, Isolation Status (n=440,374) | 25,624/414,750 | 4,814/435,560 |
| High, not isolated (n=251,636) | 12,701/238,935 | 2,244/249,392 |
| Moderate, not isolated (n=71,336) | 3,893/67,443 | 707/70,629 |
| Low, not isolated (n=77,904) | 5,357/72,547 | 1,030/76,874 |
| High, isolated (n=20,596) | 1,769/18,827 | 405/20,191 |
| Moderate, isolated (n=7,493) | 672/6,821 | 136/7,357 |
| Low, isolated (n=11,409) | 1,232/10,177 | 292/11,117 |
| Sedentary Behaviour, Loneliness Status (n=473,961) | 28,666/445,295 | 5,444/468,517 |
| Low, not lonely (n=154,521) | 7,298/147,223 | 1,252/153,269 |
| Moderate, not lonely (n=162,399) | 9,527/152,872 | 1,712/160,687 |
| High, not lonely (n=126,919) | 9,406/117,513 | 1,912/125,007 |
| Low, lonely (n=8,471) | 570/7,901 | 134/8,337 |
| Moderate, lonely (n=10,298) | 785/9,513 | 160/10,138 |
| High, lonely (n=11,353) | 1,080/10,273 | 274/11,079 |
| Sedentary Behaviour, Isolation Status (n=487,594) | 29,278/458,316 | 5,562/482,032 |
| Low, not isolated (n=153,032) | 6,879/146,153 | 1,172/151,860 |
| Moderate, not isolated (n=162,933) | 9,271/153,662 | 1,662/161,271 |
| High, not isolated (n=126,904) | 8,856/118,048 | 1,741/125,163 |
| Low, isolated (n=14,172) | 1,126/13,046 | 246/13,926 |
| Moderate, isolated (n=14,856) | 1,311/13,545 | 274/14,582 |
| High, isolated (n=15,697) | 1,835/13,862 | 467/15,230 |

a. Physical activity levels were categorized based on public health guidelines: low active (<600 MET-mins/week), moderate active (600<1200) and high active (≥1200 MET-mins/week). Sedentary behaviour was categorized into: low, ≤3.5 hours/day; moderate, 3.5≤5.5 hours/day; high >5.5 hours/day.

**Supplementary Table S4.** Number of major non-fatal CVD events by joint category of social health status and movement behaviour

| Joint Category ^a^ | N With/Without Events |
| --- | --- |
|  | Major non-fatal cardiovascular event |
| Physical Activity, Loneliness Status (n=403263) | 40,066/363,197 |
| High, not lonely (n=236,824) | 22,943/213,881 |
| Moderate, not lonely (n=67,777) | 6,397/61,380 |
| Low, not lonely (n=74,274) | 7,854/66,420 |
| High, lonely (n=13,869) | 1,549/12,320 |
| Moderate, lonely (n=4,236) | 502/3,734 |
| Low, lonely (n=6,283) | 821/5,462 |
| Physical Activity, Isolation Status (n=415,137) | 41,203/373,934 |
| High, not isolated (n=238,407) | 23,067/215,340 |
| Moderate, not isolated (n=67,403) | 6,410/60,993 |
| Low, not isolated (n=72,638) | 7,664/64,974 |
| High, isolated (n=19,287) | 2,089/17,198 |
| Moderate, isolated (n=6,981) | 738/6,243 |
| Low, isolated (n=10,421) | 1,235/9,186 |
| Sedentary Behaviour, Loneliness Status (n=446,325) | 44,994/401,331 |
| Low, not lonely (n=148,190) | 11,884/136,306 |
| Moderate, not lonely (n=153,209) | 15,700/137,509 |
| High, not lonely (n=117,178) | 14,122/103,056 |
| Low, lonely (n=7,962) | 775/7,187 |
| Moderate, lonely (n=9,555) | 1,100/8,455 |
| High, lonely (n=10,231) | 1,413/8,818 |
| Sedentary Behaviour, Isolation Status (n=459,110) | 46,146/412,964 |
| Low, not isolated (n=146,742) | 11,724/135,018 |
| Moderate, not isolated (n=153,701) | 15,705/137,996 |
| High, not isolated (n=117,200) | 14,079/103,121 |
| Low, isolated (n=13,381) | 1,256/12,125 |
| Moderate, isolated (n=13,857) | 1,521/12,336 |
| High, isolated (n=14,229) | 1,861/12,368 |

^a^ Physical activity levels were categorized based on public health guidelines: low active (<600 MET-mins/week), moderate active (600<1200) and high active (≥1200 MET-mins/week). Sedentary behaviour was categorized into: low, ≤3.5 hours/day; moderate, 3.5≤5.5 hours/day; high >5.5 hours/day.

**Supplementary Table S5a.** Independent effects of physical activity, sedentary behaviour, loneliness and social isolation on all-cause and CVD mortality and CVD events

|  | All-cause mortality ^a^ | CVD mortality ^b^ | Major non-fatal cardiovascular event ^b,c^ |
| --- | --- | --- | --- |
|  | HR (95% CIs) | HR (95% CIs) | HR (95% CIs) |
| **Loneliness** ^d^ |  |  |  |
| Not lonely (best) | ref | ref | ref |
| Lonely | 0.98 (0.94-1.03) | 1.19 (1.07-1.32) ** | 1.04 (1.00-1.08) |
| **Social isolation** ^e^ |  |  |  |
| Not isolated (best) | ref | ref | ref |
| Isolated | 1.33 (1.28-1.38) *** | 1.49 (1.37-1.62) *** | 1.00 (0.96-1.03) |
| **Physical Activity** ^f^ |  |  |  |
| High (best) | ref | ref | ref |
| Moderate | 1.09 (1.05-1.13) *** | 1.08 (1.00-1.17) | 0.98 (0.96-1.01) |
| Low | 1.24 (1.21-1.28) *** | 1.23 (1.15-1.33) *** | 1.03 (1.00-1.06) * |
| **Sedentary Behaviour** ^g^ |  |  |  |
| Low (best) | ref | ref | ref |
| Moderate | 1.05 (1.01-1.08) ** | 1.00 (0.92-1.08) | 1.07 (1.04-1.10) *** |
| High | 1.08 (1.05-1.12) *** | 1.02 (0.94-1.10) | 1.09 (1.06-1.12) *** |

^a^ adjusted for age, sex, ethnicity, smoking status, alcohol consumption, education, socioeconomic status, depressive symptoms, diabetes, CVD and cancer

^b^ adjusted for age, sex, ethnicity, smoking status, alcohol consumption, education, socioeconomic status, depressive symptoms, diabetes, hypertension, high cholesterol and BMI.

^c^ Participants with CVD at baseline were excluded.

^d^ further adjusted for isolation, physical activity and sedentary behaviour.

^e^ further adjusted for loneliness, physical activity and sedentary behaviour.

^f^ further adjusted for sedentary behaviour, loneliness and social isolation.

^g^ further adjusted for physical activity, loneliness and social isolation.

*p<0.05, **p<0.01, ***p<0.001.

**Supplementary Table S5b.** Independent effects of physical activity, sedentary behaviour, loneliness and social isolation on all-cause and CVD mortality and CVD events stratified by sex

|  | All-cause mortality ^a^ | CVD mortality ^b^ | Major non-fatal cardiovascular event ^b,c^ |
| --- | --- | --- | --- |
|  | HR (95% CIs) | HR (95% CIs) | HR (95% CIs) |
| **Men** |  |  |  |
| **Loneliness** ^d^ |  |  |  |
| Not lonely (best) | ref | ref | ref |
| Lonely | 0.94 (0.88-1.01) | 1.16 (1.01-1.33) * | 1.07 (1.01-1.13) * |
| **Social isolation** ^e^ |  |  |  |
| Not isolated (best) | ref | ref | ref |
| Isolated | 1.39 (1.32-1.46) *** | 1.56 (1.41-1.72) *** | 0.95 (0.91-1.00) * |
| **Physical Activity** ^f^ |  |  |  |
| High (best) | ref | ref | ref |
| Moderate | 1.09 (1.04-1.14) *** | 1.06 (0.96-1.17) | 0.99 (0.96-1.03) |
| Low | 1.22 (1.17-1.27) *** | 1.18 (1.08-1.28) *** | 1.02 (0.99-1.05) |
| **Sedentary Behaviour** ^g^ |  |  |  |
| Low (best) | ref | ref | ref |
| Moderate | 1.02 (0.98-1.07) | 0.99 (0.9-1.09) | 1.09 (1.05-1.12) *** |
| High | 1.04 (0.99-1.09) | 1.00 (0.91-1.1) | 1.08 (1.05-1.12) *** |
| **Women** |  |  |  |
| **Loneliness** ^d^ |  |  |  |
| Not lonely (best) | ref | ref | ref |
| Lonely | 1.04 (0.95-1.13) | 1.21 (0.97-1.52) | 1.00 (0.94-1.07) |
| **Social isolation** ^e^ |  |  |  |
| Not isolated (best) | ref | ref | ref |
| Isolated | 1.26 (1.18-1.35) *** | 1.38 (1.16-1.65) *** | 1.06 (1.00-1.11) * |
| **Physical Activity** ^f^ |  |  |  |
| High (best) | ref | ref | ref |
| Moderate | 1.09 (1.03-1.15) ** | 1.20 (1.01-1.41) * | 0.98 (0.94-1.02) |
| Low | 1.28 (1.22-1.35) *** | 1.46 (1.26-1.69) *** | 1.05 (1.01-1.10) * |
| **Sedentary Behaviour** ^g^ |  |  |  |
| Low (best) | ref | ref | ref |
| Moderate | 1.07 (1.02-1.13) ** | 1.05 (0.9-1.22) | 1.04 (1.00-1.08) * |
| High | 1.15 (1.09-1.22) *** | 1.05 (0.89-1.24) | 1.09 (1.05-1.14) *** |

^a^ adjusted for age, sex, ethnicity, smoking status, alcohol consumption, education, socioeconomic status, depressive symptoms, diabetes, CVD and cancer

^b^ adjusted for age, sex, ethnicity, smoking status, alcohol consumption, education, socioeconomic status, depressive symptoms, diabetes, hypertension, high cholesterol and BMI.

^c^ Participants with CVD at baseline were excluded.

^d^ further adjusted for isolation, physical activity and sedentary behaviour.

^e^ further adjusted for loneliness, physical activity and sedentary behaviour.

^f^ further adjusted for sedentary behaviour, loneliness and social isolation.

^g^ further adjusted for physical activity, loneliness and social isolation.

*p<0.05, **p<0.01, ***p<0.001.

**Supplementary Table S5c.** Independent effects of physical activity, sedentary behaviour, loneliness and social isolation on all-cause and CVD mortality and CVD events with multiple imputation

|  | All-cause mortality ^a^ | CVD mortality ^b^ | Major non-fatal cardiovascular event ^b,c^ |
| --- | --- | --- | --- |
|  | HR (95% CIs) | HR (95% CIs) | HR (95% CIs) |
| **Loneliness** ^d^ |  |  |  |
| Not lonely (best) | ref | ref | ref |
| Lonely | 1.00 (0.95-1.04) | 1.16 (1.06-1.25) ** | 1.02 (0.98-1.06) |
| **Social isolation** ^e^ |  |  |  |
| Not isolated (best) | ref | ref | ref |
| Isolated | 1.30 (1.26-1.33) *** | 1.47 (1.40-1.54) *** | 0.99 (0.95-1.02) |
| **Physical Activity** ^f^ |  |  |  |
| High (best) | ref | ref | ref |
| Moderate | 1.10 (1.06-1.13) *** | 1.09 (1.02-1.16) * | 1.01 (0.98-1.03) |
| Low | 1.25 (1.22-1.28) *** | 1.24 (1.18-1.30) *** | 1.07 (1.05-1.09) *** |
| **Sedentary Behaviour** ^g^ |  |  |  |
| Low (best) | ref | ref | ref |
| Moderate | 1.03 (1.00-1.06) * | 1 .00 (0.93-1.06) | 1.10 (1.07-1.12) *** |
| High | 1.07 (1.04-1.10) *** | 1.01 (0.94-1.08) | 1.15 (1.12-1.17) *** |

^a^ adjusted for age, sex, ethnicity, smoking status, alcohol consumption, education, socioeconomic status, depressive symptoms, diabetes, CVD and cancer

^b^ adjusted for age, sex, ethnicity, smoking status, alcohol consumption, education, socioeconomic status, depressive symptoms, diabetes, hypertension, high cholesterol and BMI.

^c^ Participants with CVD at baseline were excluded.

^d^ further adjusted for isolation, physical activity and sedentary behaviour.

^e^ further adjusted for loneliness, physical activity and sedentary behaviour.

^f^ further adjusted for sedentary behaviour, loneliness and social isolation.

^g^ further adjusted for physical activity, loneliness and social isolation.

*p<0.05, **p<0.01, ***p<0.001.

**Supplementary Table S6a.** Joint associations of movement behaviour and social health status with all-cause mortality

| Joint category ^a^ | Model 1  HR (95% CIs) | Model 2 ^b^  HR (95% CIs) | Model 3 ^c^  HR (95% CIs) |
| --- | --- | --- | --- |
| Physical Activity, Loneliness Status | | | |
| High, not lonely | ref | ref | ref |
| Moderate, not lonely | 1.08 (1.05-1.12) *** | 1.06 (1.03-1.10) *** | 1.09 (1.05-1.13) *** |
| Low, not lonely | 1.38 (1.34-1.42) *** | 1.29 (1.25-1.33) *** | 1.25 (1.21-1.29) *** |
| High, lonely | 1.28 (1.20-1.36) *** | 1.14 (1.07-1.22) *** | 0.99 (0.93-1.06) |
| Moderate, lonely | 1.54 (1.39-1.71) *** | 1.31 (1.17-1.45) *** | 1.12 (1.01-1.25) * |
| Low, lonely | 1.88 (1.74-2.03) *** | 1.50 (1.38-1.62) *** | 1.18 (1.09-1.28) *** |
| Physical Activity, Isolation Status | | | |
| High, not isolated | ref | ref | ref |
| Moderate, not isolated | 1.08 (1.04-1.12) *** | 1.07 (1.04-1.11) *** | 1.09 (1.05-1.13) *** |
| Low, not isolated | 1.37 (1.32-1.41) *** | 1.31 (1.27-1.35) *** | 1.26 (1.22-1.30) *** |
| High, isolated | 1.74 (1.66-1.83) *** | 1.72 (1.63-1.81) *** | 1.36 (1.30-1.44) *** |
| Moderate, isolated | 1.82 (1.69-1.97) *** | 1.77 (1.63-1.91) *** | 1.47 (1.36-1.59) *** |
| Low, isolated | 2.21 (2.08-2.34) *** | 2.04 (1.92-2.17) *** | 1.58 (1.49-1.68) *** |
| Sedentary Behaviour, Loneliness Status | | | |
| Low, not lonely | ref | ref | ref |
| Moderate, not lonely | 1.26 (1.22-1.30) *** | 1.27 (1.23-1.31) *** | 1.05 (1.01-1.08) ** |
| High, not lonely | 1.61 (1.57-1.66) *** | 1.58 (1.53-1.63) *** | 1.09 (1.05-1.13) *** |
| Low, lonely | 1.44 (1.32-1.57) *** | 1.26 (1.14-1.39) *** | 1.02 (0.93-1.13) |
| Moderate, lonely | 1.64 (1.53-1.77) *** | 1.47 (1.35-1.59) *** | 1.05 (0.96-1.14) |
| High, lonely | 2.08 (1.95-2.22) *** | 1.77 (1.65-1.90) *** | 1.04 (0.96-1.12) |
| Sedentary Behaviour, Isolation Status | | | |
| Low, not isolated | ref | ref | ref |
| Moderate, not isolated | 1.28 (1.24-1.32) *** | 1.29 (1.25-1.33) *** | 1.05 (1.02-1.09) ** |
| High, not isolated | 1.59 (1.54-1.64) *** | 1.58 (1.52-1.63) *** | 1.08 (1.04-1.12) *** |
| Low, isolated | 1.81 (1.70-1.93) *** | 1.77 (1.65-1.90) *** | 1.34 (1.24-1.43) *** |
| Moderate, isolated | 2.03 (1.91-2.15) *** | 1.96 (1.83-2.09) *** | 1.34 (1.25-1.43) *** |
| High, isolated | 2.74 (2.61-2.89) *** | 2.66 (2.51-2.82) *** | 1.47 (1.39-1.56) *** |

^a^ Physical activity levels were categorized based on public health guidelines: low active (<600 MET-mins/week), moderate active (600<1200) and high active (≥1200 MET-mins/week). Sedentary behaviour was categorized into: low, ≤3.5 hours/day; moderate, 3.5≤5.5 hours/day; high >5.5 hours/day.

^b^ Mutually adjusted for physical activity, sedentary behaviour, loneliness and social isolation, where applicable.

^c^ Further adjusted for age, sex, ethnicity, smoking status, alcohol consumption, education, socioeconomic status, depressive symptoms, diabetes, CVD and cancer.

*p<0.05, **p<0.01, ***p<0.001.

**Supplementary Table S6b.** Joint associations of movement behaviour and social health status with all-cause mortality in men

| Joint category ^a^ | Model 1  HR (95% CIs) | Model 2 ^b^  HR (95% CIs) | Model 3 ^c^  HR (95% CIs) |
| --- | --- | --- | --- |
| Physical Activity, Loneliness Status | | | |
| High, not lonely | ref | ref | ref |
| Moderate, not lonely | 1.12 (1.07, 1.17) *** | 1.10 (1.05, 1.15) *** | 1.10 (1.05, 1.15) *** |
| Low, not lonely | 1.40 (1.34, 1.46) *** | 1.32 (1.27, 1.37) *** | 1.23 (1.18, 1.29) *** |
| High, lonely | 1.23 (1.13, 1.33) *** | 1.09 (1.00, 1.19) * | 0.97 (0.89, 1.06) |
| Moderate, lonely | 1.52 (1.33, 1.73) *** | 1.30 (1.13, 1.49) *** | 1.06 (0.93, 1.22) |
| Low, lonely | 1.88 (1.71, 2.08) *** | 1.51 (1.36, 1.67) *** | 1.16 (1.04, 1.29) ** |
| Physical Activity, Isolation Status | | | |
| High, not isolated | ref | ref | ref |
| Moderate, not isolated | 1.11 (1.06, 1.17) *** | 1.12 (1.07, 1.17) *** | 1.10 (1.05, 1.16) *** |
| Low, not isolated | 1.40 (1.34, 1.45) *** | 1.35 (1.30, 1.41) *** | 1.26 (1.20, 1.31) *** |
| High, isolated | 1.76 (1.66, 1.87) *** | 1.77 (1.66, 1.88) *** | 1.44 (1.35, 1.54) *** |
| Moderate, isolated | 1.85 (1.67, 2.04) *** | 1.84 (1.66, 2.03) *** | 1.54 (1.39, 1.71) *** |
| Low, isolated | 2.16 (2.01, 2.33) *** | 2.10 (1.94, 2.27) *** | 1.59 (1.47, 1.73) *** |
| Sedentary Behaviour, Loneliness Status | | | |
| Low, not lonely | ref | ref | ref |
| Moderate, not lonely | 1.16 (1.12, 1.21) *** | 1.19 (1.14, 1.25) *** | 1.04 (0.99, 1.08) |
| High, not lonely | 1.35 (1.29, 1.40) *** | 1.34 (1.28, 1.40) *** | 1.05 (1.00, 1.10) * |
| Low, lonely | 1.47 (1.32, 1.65) *** | 1.26 (1.11, 1.43) *** | 1.05 (0.93, 1.20) |
| Moderate, lonely | 1.44 (1.30, 1.59) *** | 1.27 (1.14, 1.42) *** | 0.97 (0.86, 1.08) |
| High, lonely | 1.77 (1.63, 1.91) *** | 1.47 (1.35, 1.61) *** | 0.98 (0.90, 1.08) |
| Sedentary Behaviour, Isolation Status | | | |
| Low, not isolated | ref | ref | ref |
| Moderate, not isolated | 1.20 (1.15, 1.26) *** | 1.21 (1.16, 1.27) *** | 1.05 (1.00, 1.10) |
| High, not isolated | 1.36 (1.30, 1.42) *** | 1.35 (1.29, 1.41) *** | 1.06 (1.01, 1.11) * |
| Low, isolated | 1.91 (1.76, 2.07) *** | 1.87 (1.71, 2.05) *** | 1.50 (1.37, 1.65) *** |
| Moderate, isolated | 1.90 (1.76, 2.06) *** | 1.86 (1.70, 2.03) *** | 1.39 (1.27, 1.51) *** |
| High, isolated | 2.32 (2.17, 2.47) *** | 2.26 (2.10, 2.43) *** | 1.43 (1.32, 1.54) *** |

^a^ Physical activity levels were categorized based on public health guidelines: low active (<600 MET-mins/week), moderate active (600<1200) and high active (≥1200 MET-mins/week). Sedentary behaviour was categorized into: low, ≤3.5 hours/day; moderate, 3.5≤5.5 hours/day; high >5.5 hours/day.

^b^ Mutually adjusted for physical activity, sedentary behaviour, loneliness and social isolation, where applicable.

^c^ Further adjusted for age, sex, ethnicity, smoking status, alcohol consumption, education, socioeconomic status, depressive symptoms, diabetes, CVD and cancer.

*p<0.05, **p<0.01, ***p<0.001.

**Supplementary Table S6c.** Joint associations of movement behaviour and social health status with all-cause mortality in women

| Joint category ^a^ | Model 1  HR (95% CIs) | Model 2 ^b^  HR (95% CIs) | Model 3 ^c^  HR (95% CIs) |
| --- | --- | --- | --- |
| Physical Activity, Loneliness Status | | | |
| High, not lonely | ref | ref | ref |
| Moderate, not lonely | 1.09 (1.03, 1.15) ** | 1.06 (1.00, 1.12) * | 1.08 (1.02, 1.14) ** |
| Low, not lonely | 1.39 (1.33, 1.46) *** | 1.32 (1.25, 1.39) *** | 1.28 (1.22, 1.35) *** |
| High, lonely | 1.32 (1.19, 1.46) *** | 1.20 (1.08, 1.34) *** | 1.02 (0.92, 1.14) |
| Moderate, lonely | 1.58 (1.34, 1.86) *** | 1.36 (1.15, 1.61) *** | 1.22 (1.03, 1.44) * |
| Low, lonely | 1.85 (1.63, 2.10) *** | 1.53 (1.34, 1.75) *** | 1.21 (1.05, 1.38) ** |
| Physical Activity, Isolation Status | | | |
| High, not isolated | ref | ref | ref |
| Moderate, not isolated | 1.08 (1.02, 1.14) ** | 1.06 (1.00, 1.13) * | 1.08 (1.02, 1.15) ** |
| Low, not isolated | 1.37 (1.30, 1.44) *** | 1.32 (1.25, 1.39) *** | 1.27 (1.21, 1.34) *** |
| High, isolated | 1.63 (1.49, 1.77) *** | 1.58 (1.45, 1.72) *** | 1.24 (1.13, 1.35) *** |
| Moderate, isolated | 1.77 (1.56, 2.01) *** | 1.69 (1.48, 1.92) *** | 1.37 (1.20, 1.56) *** |
| Low, isolated | 2.25 (2.05, 2.47) *** | 2.04 (1.85, 2.25) *** | 1.57 (1.42, 1.73) *** |
| Sedentary Behaviour, Loneliness Status | | | |
| Low, not lonely | ref | ref | ref |
| Moderate, not lonely | 1.24 (1.18, 1.29) *** | 1.23 (1.17, 1.29) *** | 1.07 (1.02, 1.12) ** |
| High, not lonely | 1.58 (1.51, 1.66) *** | 1.51 (1.43, 1.59) *** | 1.17 (1.11, 1.23) *** |
| Low, lonely | 1.32 (1.16, 1.50) *** | 1.17 (1.01, 1.36) * | 0.97 (0.83, 1.13) |
| Moderate, lonely | 1.74 (1.56, 1.93) *** | 1.57 (1.38, 1.77) *** | 1.17 (1.03, 1.32) * |
| High, lonely | 2.01 (1.80, 2.24) *** | 1.75 (1.55, 1.98) *** | 1.13 (1.00, 1.29) |
| Sedentary Behaviour, Isolation Status | | | |
| Low, not isolated | ref | ref | ref |
| Moderate, not isolated | 1.23 (1.18, 1.29) *** | 1.23 (1.17, 1.30) *** | 1.07 (1.02, 1.12) * |
| High, not isolated | 1.50 (1.43, 1.58) *** | 1.46 (1.38, 1.54) *** | 1.13 (1.07, 1.20) *** |
| Low, isolated | 1.50 (1.36, 1.66) *** | 1.43 (1.27, 1.60) *** | 1.12 (0.99, 1.25) |
| Moderate, isolated | 1.94 (1.78, 2.13) *** | 1.84 (1.66, 2.03) *** | 1.29 (1.16, 1.43) *** |
| High, isolated | 2.73 (2.51, 2.97) *** | 2.59 (2.35, 2.85) *** | 1.56 (1.42, 1.72) *** |

^a^ Physical activity levels were categorized based on public health guidelines: low active (<600 MET-mins/week), moderate active (600<1200) and high active (≥1200 MET-mins/week). Sedentary behaviour was categorized into: low, ≤3.5 hours/day; moderate, 3.5≤5.5 hours/day; high >5.5 hours/day.

^b^ Mutually adjusted for physical activity, sedentary behaviour, loneliness and social isolation, where applicable.

^c^ Further adjusted for age, sex, ethnicity, smoking status, alcohol consumption, education, socioeconomic status, depressive symptoms, diabetes, CVD and cancer.

*p<0.05, **p<0.01, ***p<0.001.

**Supplementary Table S7a.** Joint associations of movement behaviour and social health status with CVD mortality

| Joint category ^a^ | Model 1  HR (95% CIs) | Model 2 ^b^  HR (95% CIs) | Model 3 ^c^  HR (95% CIs) |
| --- | --- | --- | --- |
| Physical Activity, Loneliness Status | | | |
| High, not lonely | ref | ref | ref |
| Moderate, not lonely | 1.11 (1.02-1.21) * | 1.07 (0.99-1.17) | 1.10 (1.01-1.20) * |
| Low, not lonely | 1.54 (1.43-1.65) *** | 1.38 (1.28-1.48) *** | 1.27 (1.18-1.37) *** |
| High, lonely | 1.76 (1.54-2.01) *** | 1.53 (1.34-1.75) *** | 1.34 (1.17-1.54) *** |
| Moderate, lonely | 1.91 (1.53-2.38) *** | 1.49 (1.18-1.87) *** | 1.23 (0.97-1.56) |
| Low, lonely | 2.44 (2.08-2.87) *** | 1.76 (1.48-2.08) *** | 1.29 (1.08-1.55) ** |
| Physical Activity, Isolation Status | | | |
| High, not isolated | ref | ref | ref |
| Moderate, not isolated | 1.11 (1.02-1.21) * | 1.10 (1.01-1.20) * | 1.11 (1.02-1.21) * |
| Low, not isolated | 1.47 (1.37-1.59) *** | 1.39 (1.29-1.50) *** | 1.26 (1.17-1.37) *** |
| High, isolated | 2.23 (2.01-2.48) *** | 2.15 (1.93-2.39) *** | 1.60 (1.43-1.79) *** |
| Moderate, isolated | 2.05 (1.72-2.44) *** | 1.93 (1.61-2.30) *** | 1.50 (1.25-1.79) *** |
| Low, isolated | 2.89 (2.56-3.26) *** | 2.55 (2.25-2.90) *** | 1.78 (1.56-2.03) *** |
| Sedentary Behaviour, Loneliness Status | | | |
| Low, not lonely | ref | ref | ref |
| Moderate, not lonely | 1.31 (1.22-1.41) *** | 1.33 (1.23-1.44) *** | 1.01 (0.93-1.10) |
| High, not lonely | 1.89 (1.76-2.03) *** | 1.82 (1.68-1.96) *** | 1.02 (0.94-1.11) |
| Low, lonely | 1.96 (1.64-2.34) *** | 1.66 (1.36-2.04) *** | 1.30 (1.05-1.60) * |
| Moderate, lonely | 1.93 (1.64-2.28) *** | 1.70 (1.42-2.05) *** | 1.12 (0.93-1.36) |
| High, lonely | 3.03 (2.66-3.45) *** | 2.57 (2.22-2.97) *** | 1.22 (1.04-1.43) * |
| Sedentary Behaviour, Isolation Status | | | |
| Low, not isolated | ref | ref | ref |
| Moderate, not isolated | 1.34 (1.24-1.44) *** | 1.35 (1.25-1.47) *** | 1.01 (0.93-1.10) |
| High, not isolated | 1.81 (1.68-1.95) *** | 1.81 (1.66-1.96) *** | 1.01 (0.92-1.10) |
| Low, isolated | 2.29 (2.00-2.63) *** | 2.18 (1.86-2.54) *** | 1.50 (1.28-1.76) *** |
| Moderate, isolated | 2.44 (2.14-2.78) *** | 2.29 (1.98-2.66) *** | 1.37 (1.18-1.60) *** |
| High, isolated | 3.98 (3.57-4.43) *** | 3.71 (3.29-4.19) *** | 1.59 (1.39-1.80) *** |

^a^ Physical activity levels were categorized based on public health guidelines: low active (<600 MET-mins/week), moderate active (600<1200) and high active (≥1200 MET-mins/week). Sedentary behaviour was categorized into: low, ≤3.5 hours/day; moderate, 3.5≤5.5 hours/day; high >5.5 hours/day.

^b^ Mutually adjusted for physical activity, sedentary behaviour, loneliness and social isolation, where applicable.

^c^ Further adjusted for age, sex, ethnicity, smoking status, alcohol consumption, education, socioeconomic status, depressive symptoms, diabetes, hypertension, high cholesterol and BMI.

*p<0.05, **p<0.01, ***p<0.001.

**Supplementary Table S7b.** Joint associations of movement behaviour and social health status with CVD mortality in men

| Joint category ^a^ | Model 1  HR (95% CIs) | Model 2 ^b^  HR (95% CIs) | Model 3 ^c^  HR (95% CIs) |
| --- | --- | --- | --- |
| Physical Activity, Loneliness Status | | | |
| High, not lonely | ref | ref | ref |
| Moderate, not lonely | 1.14 (1.04, 1.26) ** | 1.11 (1.01, 1.22) * | 1.09 (0.99, 1.20) |
| Low, not lonely | 1.53 (1.41, 1.66) *** | 1.40 (1.29, 1.53) *** | 1.23 (1.13, 1.34) *** |
| High, lonely | 1.70 (1.46, 1.97) *** | 1.49 (1.28, 1.74) *** | 1.36 (1.16, 1.60) *** |
| Moderate, lonely | 1.67 (1.28, 2.18) *** | 1.38 (1.05, 1.81) * | 1.12 (0.85, 1.49) |
| Low, lonely | 2.31 (1.92, 2.79) *** | 1.70 (1.39, 2.07) *** | 1.24 (1.00, 1.53) |
| Physical Activity, Isolation Status | | | |
| High, not isolated | ref | ref | ref |
| Moderate, not isolated | 1.14 (1.03, 1.26) ** | 1.14 (1.03, 1.26) ** | 1.10 (0.99, 1.22) |
| Low, not isolated | 1.45 (1.33, 1.58) *** | 1.40 (1.28, 1.53) *** | 1.21 (1.10, 1.32) *** |
| High, isolated | 2.16 (1.92, 2.43) *** | 2.12 (1.87, 2.40) *** | 1.65 (1.45, 1.87) *** |
| Moderate, isolated | 1.90 (1.55, 2.33) *** | 1.85 (1.50, 2.28) *** | 1.45 (1.17, 1.80) *** |
| Low, isolated | 2.78 (2.42, 3.21) *** | 2.62 (2.27, 3.04) *** | 1.83 (1.58, 2.14) *** |
| Sedentary Behaviour, Loneliness Status | | | |
| Low, not lonely | ref | ref | ref |
| Moderate, not lonely | 1.15 (1.05, 1.25) ** | 1.20 (1.09, 1.32) *** | 1.01 (0.92, 1.11) |
| High, not lonely | 1.41 (1.29, 1.53) *** | 1.38 (1.26, 1.52) *** | 1.01 (0.92, 1.12) |
| Low, lonely | 1.86 (1.50, 2.30) *** | 1.56 (1.23, 1.98) *** | 1.34 (1.05, 1.72) * |
| Moderate, lonely | 1.60 (1.31, 1.96) *** | 1.41 (1.13, 1.76) ** | 1.07 (0.85, 1.35) |
| High, lonely | 2.31 (1.99, 2.69) *** | 1.92 (1.62, 2.26) *** | 1.21 (1.01, 1.45) * |
| Sedentary Behaviour, Isolation Status | | | |
| Low, not isolated | ref | ref | ref |
| Moderate, not isolated | 1.17 (1.07, 1.29) *** | 1.19 (1.08, 1.32) *** | 1.00 (0.91, 1.11) |
| High, not isolated | 1.37 (1.25, 1.49) *** | 1.36 (1.24, 1.50) *** | 1.00 (0.90, 1.11) |
| Low, isolated | 2.14 (1.82, 2.52) *** | 2.01 (1.68, 2.41) *** | 1.58 (1.31, 1.89) *** |
| Moderate, isolated | 2.16 (1.85, 2.53) *** | 2.07 (1.74, 2.46) *** | 1.45 (1.22, 1.73) *** |
| High, isolated | 3.00 (2.65, 3.41) *** | 2.81 (2.44, 3.23) *** | 1.60 (1.38, 1.85) *** |

^a^ Physical activity levels were categorized based on public health guidelines: low active (<600 MET-mins/week), moderate active (600<1200) and high active (≥1200 MET-mins/week). Sedentary behaviour was categorized into: low, ≤3.5 hours/day; moderate, 3.5≤5.5 hours/day; high >5.5 hours/day.

^b^ Mutually adjusted for physical activity, sedentary behaviour, loneliness and social isolation, where applicable.

^c^ Further adjusted for age, sex, ethnicity, smoking status, alcohol consumption, education, socioeconomic status, depressive symptoms, diabetes, hypertension, high cholesterol and BMI.

*p<0.05, **p<0.01, ***p<0.001.

**Supplementary Table S7c.** Joint associations of movement behaviour and social health status with CVD mortality in women

| Joint category ^a^ | Model 1  HR (95% CIs) | Model 2 ^b^  HR (95% CIs) | Model 3 ^c^  HR (95% CIs) |
| --- | --- | --- | --- |
| Physical Activity, Loneliness Status | | | |
| High, not lonely | ref | ref | ref |
| Moderate, not lonely | 1.18 (1.01, 1.39) * | 1.14 (0.96, 1.34) | 1.15 (0.98, 1.36) |
| Low, not lonely | 1.71 (1.49, 1.97) *** | 1.58 (1.38, 1.82) *** | 1.43 (1.24, 1.65) *** |
| High, lonely | 1.78 (1.35, 2.35) *** | 1.58 (1.19, 2.09) ** | 1.27 (0.95, 1.70) |
| Moderate, lonely | 2.69 (1.83, 3.96) *** | 2.00 (1.31, 3.03) ** | 1.56 (1.02, 2.40) * |
| Low, lonely | 2.75 (2.00, 3.77) *** | 2.14 (1.53, 2.99) *** | 1.49 (1.06, 2.10) * |
| Physical Activity, Isolation Status | | | |
| High, not isolated | ref | ref | ref |
| Moderate, not isolated | 1.18 (1.00, 1.39) | 1.15 (0.97, 1.37) | 1.16 (0.98, 1.38) |
| Low, not isolated | 1.69 (1.47, 1.95) *** | 1.62 (1.40, 1.88) *** | 1.46 (1.26, 1.70) *** |
| High, isolated | 2.08 (1.65, 2.62) *** | 2.03 (1.60, 2.56) *** | 1.45 (1.14, 1.84) ** |
| Moderate, isolated | 2.45 (1.76, 3.40) *** | 2.26 (1.61, 3.17) *** | 1.66 (1.18, 2.33) ** |
| Low, isolated | 3.09 (2.42, 3.95) *** | 2.62 (2.02, 3.40) *** | 1.65 (1.25, 2.17) *** |
| Sedentary Behaviour, Loneliness Status | | | |
| Low, not lonely | ref | ref | ref |
| Moderate, not lonely | 1.28 (1.13, 1.46) *** | 1.22 (1.06, 1.41) ** | 1.00 (0.86, 1.16) |
| High, not lonely | 1.75 (1.53, 2.01) *** | 1.57 (1.34, 1.83) *** | 1.05 (0.89, 1.24) |
| Low, lonely | 1.84 (1.33, 2.54) *** | 1.52 (1.03, 2.24) * | 1.17 (0.79, 1.73) |
| Moderate, lonely | 2.14 (1.61, 2.85) *** | 1.87 (1.34, 2.60) *** | 1.24 (0.88, 1.73) |
| High, lonely | 2.69 (2.03, 3.55) *** | 2.39 (1.75, 3.26) *** | 1.25 (0.90, 1.74) |
| Sedentary Behaviour, Isolation Status | | | |
| Low, not isolated | ref | ref | ref |
| Moderate, not isolated | 1.29 (1.13, 1.47) *** | 1.25 (1.08, 1.45) ** | 1.03 (0.88, 1.20) |
| High, not isolated | 1.59 (1.38, 1.83) *** | 1.50 (1.28, 1.77) *** | 1.02 (0.86, 1.21) |
| Low, isolated | 1.92 (1.48, 2.50) *** | 1.81 (1.34, 2.46) *** | 1.33 (0.98, 1.81) |
| Moderate, isolated | 2.23 (1.74, 2.84) *** | 1.92 (1.44, 2.57) *** | 1.15 (0.85, 1.55) |
| High, isolated | 3.80 (3.08, 4.70) *** | 3.34 (2.61, 4.28) *** | 1.55 (1.19, 2.03) ** |

^a^ Physical activity levels were categorized based on public health guidelines: low active (<600 MET-mins/week), moderate active (600<1200) and high active (≥1200 MET-mins/week). Sedentary behaviour was categorized into: low, ≤3.5 hours/day; moderate, 3.5≤5.5 hours/day; high >5.5 hours/day.

^b^ Mutually adjusted for physical activity, sedentary behaviour, loneliness and social isolation, where applicable.

^c^ Further adjusted for age, sex, ethnicity, smoking status, alcohol consumption, education, socioeconomic status, depressive symptoms, diabetes, hypertension, high cholesterol and BMI.

*p<0.05, **p<0.01, ***p<0.001.

**Supplementary Table S8a.** Joint associations of movement behaviour and social health status with major non-fatal cardiovascular events

| Joint category ^a^ | Model 1  HR (95% CIs) | Model 2 ^b^  HR (95% CIs) | Model 3 ^c^  HR (95% CIs) |
| --- | --- | --- | --- |
| Physical Activity, Loneliness Status | | | |
| High, not lonely | ref | ref | ref |
| Moderate, not lonely | 0.97 (0.95-1.00) | 0.96 (0.94-0.99) * | 0.98 (0.95-1.01) |
| Low, not lonely | 1.10 (1.07-1.13) *** | 1.05 (1.03-1.08) *** | 1.03 (1.00-1.06) * |
| High, lonely | 1.17 (1.11-1.23) *** | 1.12 (1.06-1.17) *** | 1.02 (0.97-1.07) |
| Moderate, lonely | 1.25 (1.14-1.36) *** | 1.17 (1.07-1.28) *** | 1.06 (0.97-1.17) |
| Low, lonely | 1.39 (1.30-1.49) *** | 1.26 (1.18-1.36) *** | 1.09 (1.01-1.17) * |
| Physical Activity, Isolation Status | | | |
| High, not isolated | ref | ref | ref |
| Moderate, not isolated | 0.98 (0.96-1.01) | 0.97 (0.94-1.00) * | 0.98 (0.95-1.01) |
| Low, not isolated | 1.10 (1.07-1.13) *** | 1.06 (1.03-1.09) *** | 1.03 (1.00-1.06) |
| High, isolated | 1.14 (1.09-1.20) *** | 1.12 (1.07-1.17) *** | 0.99 (0.94-1.03) |
| Moderate, isolated | 1.12 (1.04-1.21) ** | 1.08 (1.00-1.17) * | 0.98 (0.91-1.06) |
| Low, isolated | 1.26 (1.19-1.34) *** | 1.17 (1.11-1.24) *** | 1.04 (0.98-1.11) |
| Sedentary Behaviour, Loneliness Status | | | |
| Low, not lonely | ref | ref | ref |
| Moderate, not lonely | 1.30 (1.27-1.33) *** | 1.31 (1.27-1.34) *** | 1.07 (1.04-1.10) *** |
| High, not lonely | 1.56 (1.52-1.60) *** | 1.56 (1.52-1.60) *** | 1.08 (1.06-1.11) *** |
| Low, lonely | 1.23 (1.15-1.33) *** | 1.21 (1.11-1.31) *** | 1.02 (0.94-1.11) |
| Moderate, lonely | 1.48 (1.39-1.57) *** | 1.48 (1.39-1.59) *** | 1.12 (1.05-1.20) ** |
| High, lonely | 1.81 (1.71-1.91) *** | 1.78 (1.67-1.89) *** | 1.13 (1.07-1.21) *** |
| Sedentary Behaviour, Isolation Status | | | |
| Low, not isolated | ref | ref | ref |
| Moderate, not isolated | 1.30 (1.27-1.33) *** | 1.31 (1.28-1.35) *** | 1.07 (1.04-1.10) *** |
| High, not isolated | 1.55 (1.51-1.59) *** | 1.56 (1.52-1.60) *** | 1.08 (1.05-1.11) *** |
| Low, isolated | 1.20 (1.13-1.27) *** | 1.18 (1.10-1.26) *** | 0.98 (0.92-1.05) |
| Moderate, isolated | 1.42 (1.35-1.50) *** | 1.41 (1.33-1.49) *** | 1.06 (1.00-1.12) |
| High, isolated | 1.74 (1.65-1.82) *** | 1.73 (1.64-1.83) *** | 1.10 (1.04-1.16) *** |

^a^ Physical activity levels were categorized based on public health guidelines: low active (<600 MET-mins/week), moderate active (600<1200) and high active (≥1200 MET-mins/week). Sedentary behaviour was categorized into: low, ≤3.5 hours/day; moderate, 3.5≤5.5 hours/day; high >5.5 hours/day.

^b^ Mutually adjusted for physical activity, sedentary behaviour, loneliness and social isolation, where applicable.

^c^ Further adjusted for age, sex, ethnicity, smoking status, alcohol consumption, education, socioeconomic status, depressive symptoms, diabetes, hypertension, high cholesterol and BMI.

*p<0.05, **p<0.01, ***p<0.001.

**Supplementary Table S8b.** Joint associations of movement behaviour and social health status with major non-fatal cardiovascular events in men

| Joint category ^a^ | Model 1  HR (95% CIs) | Model 2 ^b^  HR (95% CIs) | Model 3 ^c^  HR (95% CIs) |
| --- | --- | --- | --- |
| Physical Activity, Loneliness Status | | | |
| High, not lonely | ref | ref | ref |
| Moderate, not lonely | 0.99 (0.96, 1.03) | 0.99 (0.96, 1.03) | 0.98 (0.95, 1.02) |
| Low, not lonely | 1.09 (1.06, 1.13) *** | 1.07 (1.03, 1.10) *** | 1.01 (0.98, 1.05) |
| High, lonely | 1.09 (1.02, 1.16) * | 1.07 (1.01, 1.15) * | 1.03 (0.96, 1.10) |
| Moderate, lonely | 1.30 (1.16, 1.45) *** | 1.27 (1.13, 1.42) *** | 1.16 (1.04, 1.31) ** |
| Low, lonely | 1.29 (1.18, 1.42) *** | 1.25 (1.14, 1.37) *** | 1.10 (1.00, 1.21) |
| Physical Activity, Isolation Status | | | |
| High, not isolated | ref | ref | ref |
| Moderate, not isolated | 1.01 (0.98, 1.05) | 1.00 (0.97, 1.04) | 0.99 (0.95, 1.03) |
| Low, not isolated | 1.10 (1.06, 1.14) *** | 1.07 (1.04, 1.11) *** | 1.01 (0.98, 1.05) |
| High, isolated | 1.02 (0.96, 1.08) | 1.02 (0.96, 1.08) | 0.94 (0.89, 1.00) |
| Moderate, isolated | 1.02 (0.92, 1.12) | 1.01 (0.92, 1.11) | 0.95 (0.86, 1.04) |
| Low, isolated | 1.14 (1.06, 1.23) *** | 1.09 (1.01, 1.18) * | 0.99 (0.92, 1.07) |
| Sedentary Behaviour, Loneliness Status | | | |
| Low, not lonely | ref | ref | ref |
| Moderate, not lonely | 1.24 (1.20, 1.28) *** | 1.24 (1.20, 1.28) *** | 1.09 (1.06, 1.13) *** |
| High, not lonely | 1.31 (1.27, 1.35) *** | 1.31 (1.26, 1.35) *** | 1.09 (1.05, 1.13) *** |
| Low, lonely | 1.24 (1.13, 1.37) *** | 1.23 (1.11, 1.37) *** | 1.12 (1.01, 1.25) * |
| Moderate, lonely | 1.29 (1.19, 1.41) *** | 1.31 (1.20, 1.43) *** | 1.11 (1.02, 1.22) * |
| High, lonely | 1.49 (1.39, 1.60) *** | 1.50 (1.39, 1.61) *** | 1.17 (1.09, 1.27) *** |
| Sedentary Behaviour, Isolation Status | | | |
| Low, not isolated | ref | ref | ref |
| Moderate, not isolated | 1.23 (1.19, 1.27) *** | 1.24 (1.19, 1.28) *** | 1.09 (1.05, 1.13) *** |
| High, not isolated | 1.30 (1.26, 1.34) *** | 1.30 (1.26, 1.35) *** | 1.08 (1.04, 1.12) *** |
| Low, isolated | 1.07 (0.99, 1.16) | 1.04 (0.95, 1.13) | 0.94 (0.86, 1.03) |
| Moderate, isolated | 1.18 (1.10, 1.27) *** | 1.19 (1.10, 1.29) *** | 1.01 (0.94, 1.10) |
| High, isolated | 1.37 (1.29, 1.46) *** | 1.35 (1.26, 1.45) *** | 1.06 (0.99, 1.13) |

^a^ Physical activity levels were categorized based on public health guidelines: low active (<600 MET-mins/week), moderate active (600<1200) and high active (≥1200 MET-mins/week). Sedentary behaviour was categorized into: low, ≤3.5 hours/day; moderate, 3.5≤5.5 hours/day; high >5.5 hours/day.

^b^ Mutually adjusted for physical activity, sedentary behaviour, loneliness and social isolation, where applicable.

^c^ Further adjusted for age, sex, ethnicity, smoking status, alcohol consumption, education, socioeconomic status, depressive symptoms, diabetes, hypertension, high cholesterol and BMI.

*p<0.05, **p<0.01, ***p<0.001.

**Supplementary Table S8c.** Joint associations of movement behaviour and social health status with major non-fatal cardiovascular events in women

| Joint category ^a^ | Model 1  HR (95% CIs) | Model 2 ^b^  HR (95% CIs) | Model 3 ^c^  HR (95% CIs) |
| --- | --- | --- | --- |
| Physical Activity, Loneliness Status | | | |
| High, not lonely | ref | ref | ref |
| Moderate, not lonely | 1.01 (0.96, 1.05) | 0.99 (0.95, 1.03) | 0.98 (0.94, 1.03) |
| Low, not lonely | 1.16 (1.11, 1.21) *** | 1.11 (1.07, 1.16) *** | 1.05 (1.01, 1.10) * |
| High, lonely | 1.25 (1.15, 1.36) *** | 1.19 (1.09, 1.29) *** | 1.01 (0.93, 1.10) |
| Moderate, lonely | 1.18 (1.02, 1.37) * | 1.08 (0.93, 1.26) | 0.92 (0.79, 1.07) |
| Low, lonely | 1.55 (1.39, 1.73) *** | 1.39 (1.24, 1.55) *** | 1.08 (0.96, 1.21) |
| Physical Activity, Isolation Status | | | |
| High, not isolated | ref | ref | ref |
| Moderate, not isolated | 1.00 (0.96, 1.05) | 0.98 (0.94, 1.03) | 0.98 (0.93, 1.02) |
| Low, not isolated | 1.15 (1.11, 1.20) *** | 1.12 (1.07, 1.17) *** | 1.05 (1.01, 1.10) * |
| High, isolated | 1.30 (1.21, 1.40) *** | 1.26 (1.17, 1.35) *** | 1.05 (0.98, 1.13) |
| Moderate, isolated | 1.28 (1.14, 1.44) *** | 1.22 (1.08, 1.37) *** | 1.03 (0.92, 1.16) |
| Low, isolated | 1.46 (1.34, 1.60) *** | 1.37 (1.25, 1.50) *** | 1.11 (1.02, 1.22) * |
| Sedentary Behaviour, Loneliness Status | | | |
| Low, not lonely | ref | ref | ref |
| Moderate, not lonely | 1.22 (1.18, 1.27) *** | 1.22 (1.17, 1.26) *** | 1.03 (0.99, 1.07) |
| High, not lonely | 1.45 (1.40, 1.51) *** | 1.43 (1.37, 1.49) *** | 1.09 (1.04, 1.13) *** |
| Low, lonely | 1.14 (1.03, 1.28) * | 1.10 (0.98, 1.25) | 0.90 (0.80, 1.02) |
| Moderate, lonely | 1.56 (1.42, 1.70) *** | 1.53 (1.39, 1.69) *** | 1.11 (1.01, 1.23) * |
| High, lonely | 1.81 (1.65, 1.99) *** | 1.69 (1.53, 1.87) *** | 1.08 (0.97, 1.20) |
| Sedentary Behaviour, Isolation Status | | | |
| Low, not isolated | ref | ref | ref |
| Moderate, not isolated | 1.22 (1.18, 1.27) *** | 1.23 (1.18, 1.27) *** | 1.04 (1.00, 1.08) |
| High, not isolated | 1.43 (1.37, 1.48) *** | 1.42 (1.36, 1.48) *** | 1.08 (1.04, 1.13) *** |
| Low, isolated | 1.22 (1.12, 1.33) *** | 1.21 (1.10, 1.33) *** | 1.02 (0.93, 1.13) |
| Moderate, isolated | 1.55 (1.44, 1.68) *** | 1.59 (1.37, 1.64) *** | 1.09 (1.00, 1.19) |
| High, isolated | 1.85 (1.71, 2.00) *** | 1.85 (1.69, 2.01) *** | 1.18 (1.08, 1.29) *** |

^a^ Physical activity levels were categorized based on public health guidelines: low active (<600 MET-mins/week), moderate active (600<1200) and high active (≥1200 MET-mins/week). Sedentary behaviour was categorized into: low, ≤3.5 hours/day; moderate, 3.5≤5.5 hours/day; high >5.5 hours/day.

^b^ Mutually adjusted for physical activity, sedentary behaviour, loneliness and social isolation, where applicable.

^c^ Further adjusted for age, sex, ethnicity, smoking status, alcohol consumption, education, socioeconomic status, depressive symptoms, diabetes, hypertension, high cholesterol and BMI.

*p<0.05, **p<0.01, ***p<0.001.

**Supplementary Table S9a.** Analyses on interaction of physical activity^a^ and loneliness with risks for all-cause mortality, CVD mortality, and major non-fatal cardiovascular disease

|  | Hazard Ratio (95% CI) | | Hazard Ratio (95% CI) for PA Scores within Strata of Loneliness |
| --- | --- | --- | --- |
|  | High PA | Mod/Low PA |  |
| **All-cause mortality**^b^ |  |  |  |
| Not lonely | 1.00 | 1.21 (1.18-1.24) | 1.21 (1.18-1.25) |
| Lonely | 1.11 (1.04-1.19) | 1.36 (1.28-1.46) | 1.24 (1.13-1.35) |
| Hazard Ratio^b^ (95% CI) for Loneliness within Strata of PA | 1.12 (1.05-1.20) | 1.12 (1.05-1.20) |  |
| **Cardiovascular disease mortality**^c^ |  |  |  |
| Not lonely | 1.00 | 1.25 (1.17-1.33) | 1.19 (1.12-1.27) |
| Lonely | 1.49 (1.30-1.71) | 1.53 (1.33-1.76) | 0.98 (0.81-1.19) |
| Hazard Ratio^b^ (95% CI) for Loneliness within Strata of PA | 1.36 (1.18-1.58) | 1.03 (0.88-1.21) |  |
| **Major non-fatal cardiovascular events**^c^ |  |  |  |
| Not lonely | 1.00 | 1.03 (1.01-1.05) | 1.03 (1.01-1.05) |
| Lonely | 1.13 (1.08-1.20) | 1.25 (1.18-1.32) | 1.09 (1.01-1.18) |
| Hazard Ratio^b^ (95% CI) for Loneliness within Strata of PA | 1.14 (1.08-1.20) | 1.21 (1.14-1.28) |  |

Measure of interaction on additive scale: for all-cause mortality, RERI (95% CI) = 0.05 (-0.07-0.16), AP (95% CI) = 0.03 (-0.05-0.12), and S = 1.15 (0.81-1.48); for cardiovascular disease mortality, RERI (95% CI) = -0.21 (-0.50-0.08), AP (95% CI) = -0.14 (-0.34-0.06), and S = 0.72 (0.25-1.19); for major non-fatal cardiovascular events, RERI (95% CI) = 0.09 (-0.00-0.18), AP (95% CI) = 0.07 (0.00-0.14), and S = 1.56 (1.08-2.03).

Measure of interaction on multiplicative scale: for all-cause mortality, hazard ratios (HR) (95% CI) = 1.02 (0.93-1.12), *P* = 0.697; for cardiovascular disease mortality, HR (95% CI) = 0.79 (0.65-0.96), *P* = 0.019; for major non-fatal cardiovascular events, HR (95% CI) = 1.07 (0.99-1.16), *P* = 0.076.

^a^ Physical activity levels were grouped into: high (≥1200 MET-mins/week); moderate and low (<1200).

^b^ Hazard ratios were adjusted for age, sex, ethnicity, smoking status, alcohol consumption, education, SES, depressive symptoms, diabetes, CVD and cancer.

^c^ Hazard ratios were adjusted for age, sex, ethnicity, smoking status, alcohol consumption, education, SES, depressive symptoms, diabetes, hypertension, high cholesterol and BMI.

**Supplementary Table S9b.** Analyses on interaction of physical activity^a^ and isolation with risks for all-cause mortality, CVD mortality, and major non-fatal cardiovascular disease

|  | Hazard Ratio (95% CI) | | Hazard Ratio (95% CI) for PA Scores within Strata of Isolation |
| --- | --- | --- | --- |
|  | High PA | Mod/Low PA |  |
| **All-cause mortality**^b^ |  |  |  |
| Not isolated | 1.00 | 1.22 (1.18-1.25) | 1.22 (1.19-1.26) |
| Isolated | 1.50 (1.42-1.58) | 1.76 (1.67-1.85) | 1.18 (1.10-1.26) |
| Hazard Ratio^b^ (95% CI) for Isolation within Strata of PA | 1.52 (1.44-1.60) | 1.42 (1.35-1.50) |  |
| **Cardiovascular disease mortality**^c^ |  |  |  |
| Not isolated | 1.00 | 1.25 (1.17-1.33) | 1.19 (1.12-1.28) |
| Isolated | 1.78 (1.60-1.99) | 1.99 (1.78-2.22) | 1.05 (0.91-1.21) |
| Hazard Ratio^b^ (95% CI) for Isolation within Strata of PA | 1.61 (1.44-1.81) | 1.38 (1.23-1.55) |  |
| **Major non-fatal cardiovascular events**^c^ |  |  |  |
| Not isolated | 1.00 | 1.03 (1.01-1.05) | 1.03 (1.01-1.05) |
| Isolated | 1.04 (0.99-1.09) | 1.10 (1.04-1.15) | 1.06 (0.99-1.13) |
| Hazard Ratio^b^ (95% CI) for Isolation within Strata of PA | 1.05 (1.00-1.10) | 1.05 (1.00-1.11) |  |

Measure of interaction on additive scale: for all-cause mortality, RERI (95% CI) = 0.04 (-0.07-0.15), AP (95% CI) = 0.02 (-0.04-0.09), and S = 1.06 (0.91-1.21); for cardiovascular disease mortality, RERI (95% CI) = -0.05 (-0.32-0.23), AP (95% CI) = -0.02 (-0.16-0.12), and S = 0.96 (0.68-1.23); for major non-fatal cardiovascular events, RERI (95% CI) = 0.03 (-0.04-0.10), AP (95% CI) = 0.03 (-0.04-0.09), and S = 1.44 (0.49-2.39).

Measure of interaction on multiplicative scale: for all-cause mortality, hazard ratios (HR) (95% CI) = 0.97 (0.90-1.04), *P* =0.350; for cardiovascular disease mortality, HR (95% CI) = 0.88 (0.75-1.03), *P* = 0.100; for major non-fatal cardiovascular events, HR (95% CI) = 1.03 (0.96-1.10), *P* = 0.455.

^a^ Physical activity levels were grouped into: high (≥1200 MET-mins/week); moderate and low (<1200).

^b^ Hazard ratios were adjusted for age, sex, ethnicity, smoking status, alcohol consumption, education, SES, depressive symptoms, diabetes, CVD and cancer.

^c^ Hazard ratios were adjusted for age, sex, ethnicity, smoking status, alcohol consumption, education, SES, depressive symptoms, diabetes, hypertension, high cholesterol and BMI.

|  | Hazard Ratio (95% CI) | | Hazard Ratio (95% CI) for SB Scores within Strata of Loneliness |
| --- | --- | --- | --- |
|  | Low Sedentary Behaviour | Mod/High Sedentary Behaviour |  |
| **All-cause mortality**^b^ |  |  |  |
| Not lonely | 1.00 | 1.12 (1.08-1.15) | 1.12 (1.08-1.15) |
| Lonely | 1.16 (1.05-1.28) | 1.24 (1.17-1.32) | 1.08 (0.97-1.20) |
| Hazard Ratio^b^ (95% CI) for Loneliness within Strata of SB | 1.16 (1.05-1.28) | 1.11 (1.05-1.17) |  |
| **Cardiovascular disease mortality**^c^ |  |  |  |
| Not lonely | 1.00 | 1.06 (0.99-1.14) | 1.01 (0.94-1.09) |
| Lonely | 1.48 (1.21-1.81) | 1.40 (1.24-1.59) | 0.96 (0.76-1.21) |
| Hazard Ratio^b^ (95% CI) for Loneliness within Strata of SB | 1.26 (1.01-1.57) | 1.17 (1.03-1.32) |  |
| **Major non-fatal cardiovascular events**^c^ |  |  |  |
| Not lonely | 1.00 | 1.10 (1.08-1.13) | 1.10 (1.08-1.13) |
| Lonely | 1.15 (1.06-1.25) | 1.30 (1.24-1.36) | 1.12 (1.03-1.23) |
| Hazard Ratio^b^ (95% CI) for Loneliness within Strata of SB | 1.15 (1.06-1.24) | 1.18 (1.12-1.23) |  |

**Supplementary Table S9c.** Analyses on interaction of sedentary behaviour^a^ and loneliness with risks for all-cause mortality, CVD mortality, and major non-fatal cardiovascular disease

Measure of interaction on additive scale: for all-cause mortality, RERI (95% CI) = -0.04 (-0.17-0.09), AP (95% CI) = -0.03 (-0.13-0.08), and S = 0.87 (0.39-1.35); for cardiovascular disease mortality, RERI (95% CI) = -0.14 (-0.47-0.19), AP (95% CI) = -0.10 (-0.34-0.14), and S = 0.75 (0.10-1.40); for major non-fatal cardiovascular events, RERI (95% CI) = 0.04 (-0.06-0.15), AP (95% CI) = 0.03 (-0.05-0.12), and S = 1.17 (0.76-1.59).

Measure of interaction on multiplicative scale: for all-cause mortality, hazard ratios (HR) (95% CI) = 0.96 (0.86-1.07), *P* = 0.450; for cardiovascular disease mortality, HR (95% CI) = 0.90 (0.71-1.13), *P* = 0.370; for major non-fatal cardiovascular events, HR (95% CI) = 1.02 (0.93-1.12), *P* = 0.635.

^a^ Sedentary behaviour was categorized into: low (≤3.5 hours/day); moderate and high (>3.5 hours/day).

^b^ Hazard ratios were adjusted for age, sex, ethnicity, smoking status, alcohol consumption, education, SES, depressive symptoms, diabetes, CVD and cancer.

^c^ Hazard ratios were adjusted for age, sex, ethnicity, smoking status, alcohol consumption, education, SES, depressive symptoms, diabetes, hypertension, high cholesterol and BMI.

**Supplementary Table S9d.** Analyses on interaction of sedentary behaviour^a^ and isolation with risks for all-cause mortality, CVD mortality, and major non-fatal cardiovascular disease

Measure of interaction on additive scale: for all-cause mortality, RERI (95% CI) = 0.06 (-0.06-0.18), AP (95% CI) = 0.04 (-0.04-0.11), and S = 1.10 (0.90-1.30); for cardiovascular disease mortality, RERI (95% CI) = 0.04 (-0.25-0.33), AP (95% CI) 0.02 (-0.14-0.19), S = 1.06 (0.67-1.44); for major non-fatal cardiovascular events, RERI (95% CI) = 0.03 (-0.05-0.11), AP (95% CI) = 0.03 (-0.04-0.10), and S = 1.25 (0.66-1.84).

Measure of interaction on multiplicative scale: for all-cause mortality, hazard ratios (HR) (95% CI) = 1.00 (0.93-1.09), *P* = 0.927; for cardiovascular disease mortality, HR (95% CI) = 0.99 (0.83-1.19), *P* = 0.930; for major non-fatal cardiovascular events, HR (95% CI) = 1.03 (0.95-1.11), *P* = 0.502.

^a^ Sedentary behaviour was categorized into: low (≤3.5 hours/day); moderate and high (>3.5 hours/day).

^b^ Hazard ratios were adjusted for age, sex, ethnicity, smoking status, alcohol consumption, education, SES, depressive symptoms, diabetes, CVD and cancer.

^c^ Hazard ratios were adjusted for age, sex, ethnicity, smoking status, alcohol consumption, education, SES, depressive symptoms, diabetes, hypertension, high cholesterol and BMI.

|  | Hazard Ratio (95% CI) | | Hazard Ratio (95% CI) for SB Scores within Strata of Isolation |
| --- | --- | --- | --- |
|  | Low Sedentary Behaviour | Mod/High Sedentary Behaviour |  |
| **All-cause mortality**^b^ |  |  |  |
| Not isolated | 1.00 | 1.11 (1.08-1.15) | 1.11 (1.08-1.15) |
| Isolated | 1.47 (1.37-1.58) | 1.64 (1.56-1.72) | 1.12 (1.03-1.20) |
| Hazard Ratio^b^ (95% CI) for Isolation within Strata of SB | 1.47 (1.37-1.58) | 1.47 (1.41-1.53) |  |
| **Cardiovascular disease mortality**^c^ |  |  |  |
| Not isolated | 1.00 | 1.05 (0.98-1.14) | 1.01 (0.93-1.09) |
| Isolated | 1.68 (1.44-1.97) | 1.77 (1.59-1.97) | 1.03 (0.86-1.22) |
| Hazard Ratio^b^ (95% CI) for Isolation within Strata of SB | 1.51 (1.28-1.77) | 1.49 (1.35-1.63) |  |
| **Major non-fatal cardiovascular events**^c^ |  |  |  |
| Not isolated | 1.00 | 1.10 (1.08-1.13) | 1.10 (1.07-1.13) |
| Isolated | 1.03 (0.97-1.10) | 1.16 (1.12-1.22) | 1.14 (1.06-1.22) |
| Hazard Ratio^b^ (95% CI) for Isolation within Strata of SB | 1.02 (0.96-1.09) | 1.06 (1.02-1.10) |  |
